# Targeting DNA repair pathway in cancer: Mechanisms and clinical application

**DOI:** 10.1002/mco2.103

**Published:** 2021-12-07

**Authors:** Manni Wang, Siyuan Chen, Danyi Ao

**Affiliations:** ^1^ Department of Biotherapy Cancer Center West China Hospital Sichuan University Chengdu China

**Keywords:** cancer, combination therapy, DNA damage response, PARP

## Abstract

Over the last decades, the growing understanding on DNA damage response (DDR) pathways has broadened the therapeutic landscape in oncology. It is becoming increasingly clear that the genomic instability of cells resulted from deficient DNA damage response contributes to the occurrence of cancer. One the other hand, these defects could also be exploited as a therapeutic opportunity, which is preferentially more deleterious in tumor cells than in normal cells. An expanding repertoire of DDR‐targeting agents has rapidly expanded to inhibitors of multiple members involved in DDR pathways, including PARP, ATM, ATR, CHK1, WEE1, and DNA‐PK. In this review, we sought to summarize the complex network of DNA repair machinery in cancer cells and discuss the underlying mechanism for the application of DDR inhibitors in cancer. With the past preclinical evidence and ongoing clinical trials, we also provide an overview of the history and current landscape of DDR inhibitors in cancer treatment, with special focus on the combination of DDR‐targeted therapies with other cancer treatment strategies.

## Graphical abstract

In this review, we sought to summarize the complex network of DNA repair machinery in cancer cells and discuss the underlying mechanism for the application of DDR inhibitors in cancer. With the past preclinical evidence and ongoing clinical trials, we also provide an overview of the history and current landscape of DDR inhibitors in cancer treatment, with especial focus on the combination of DDR‐targeted therapies with other cancer treatment strategies.

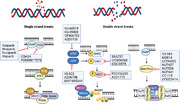

## 1 INTRODUCTION

As early as 1914, a German scientist Theodor Boveri published his work on the origin of malignant tumors, which suggested the “specific and abnormal chromosome constitution” could attribute to the onset of cancer.^1^ Through out the century, compelling data are emerging to support the role of genomic instability in cancer, including the alteration in chromosome number and structure, and moreover, in DNA compositions. These changes may lead to oncogenic transformation and confer resistance to anticancer therapies. Alongside direct damage caused by genetic alterations, some mutations have been characterized as collateral damage from the loss of genome integrity caused by carcinogens. Common oncogenic factors that result in genomic instability include chemical carcinogens in the environment, genotoxic anticancer drugs,^2^ and endogeneous carcinogens such as microbial metabolism products^3^ and free radicals produced by ionizing radiation.^4^

To limit the progression of DNA lesions, cells have evolved complex DNA repair machinery, which triggers cell‐cycle checkpoints and allows DNA damage repair before it further interferes with the replication process. Excessive DNA damage or deficient DNA repair would thus result in accumulating genomic disorders that ultimately contribute to cell death. Thus, the fate of a cell following critical DNA damage is largely decided by the amount of DNA damage and its repair capacity. On the other hand, the misrepair of single‐strand breaks (SSBs) and double‐strand breaks (DSBs) of DNA may result in genome rearrangement. The DNA repair capacity varies among different cell types, with some tumor cells exhibit significantly enhanced DNA repair following replication and genotoxic stress.^5^

In parallel with the advances in tumor biology that introduce DDR as potential therapeutic targets, a range of inhibitors targeting DDR components have emerged, some of which are now under clinical investigation. Moreover, emerging evidence suggests the sensitization effect of DDR inhibitors to conventional cancer therapies, and the correlation between DDR pathways and immune checkpoint inhibitor (ICI) response, which together encourages the design DDR inhibitor‐based combination treatments. In this review, we sought to summarize the complex network of DNA repair machinery in cancer cells and to discuss the underlying mechanism for the application of DDR inhibitors in cancer. With the past preclinical evidence and ongoing clinical trials, we especially summarized the ongoing clinicals that involve DDR inhibitors, with special focus on the combination therapy of DDR inhibitors including chemotherapy, radiotherapy, immunotherapies, and combinations DDR inhibitors, hopefully providing an overview of the history and current landscape of DDR inhibitors.

## 2 DNA DAMAGE AND THE DNA DAMAGE RESPONSE

**FIGURE 1:**
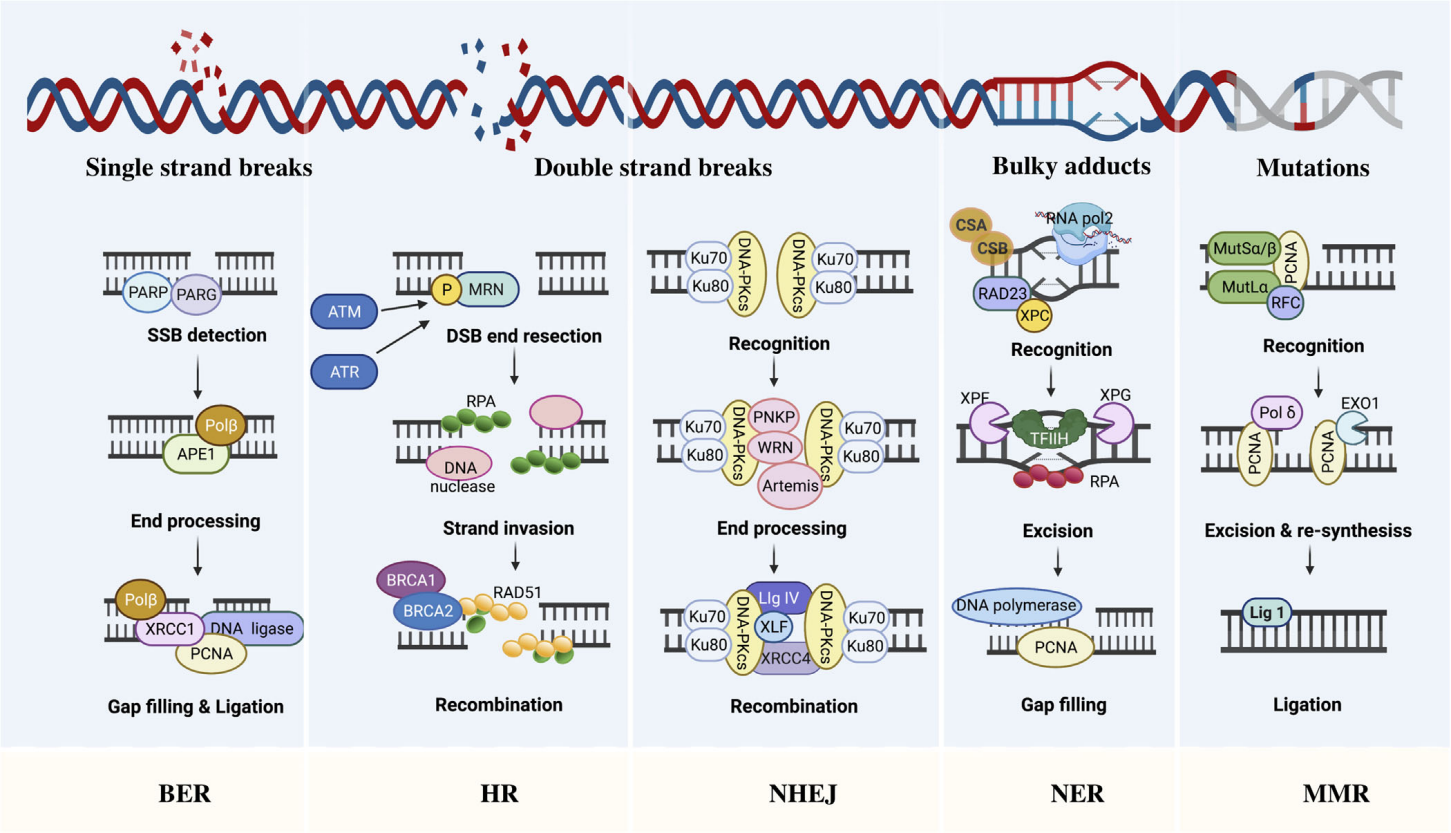
Overview of major pathways for the repair of different DNA damage. Single‐strand breaks (SSBs) are repaired by direct and indirect base excision repair (BER) and double‐strand breaks (DSBs) are repaired by homologous recombination (HR) and nonhomologous end joining (NHEJ). Replication error is repaired by mismatch repair (MMR) and DNA adducts by nucleotide excision repair (NER). Figure was created with Biorender

To maintain genomic integrity, an intricate DNA repair system is evolved to counteract various forms of DNA lesions, and these mechanisms are referred to as the DNA damage response (DDR). Here we classified DDR pathways into three functionally interwoven parts: the sensor that detects DNA damage, signal transducer that triggers signaling cascades, and effector that impedes DNA repair. Numerous efforts have been undertaken to elucidate the machinery for the repair of genotoxic lesions in mammalian cells. These pathways are not mutually exclusive processes, but rather coordinated with each other to form a precise regulation network of DNA repair. Figure 1 presents an overview of major pathways for the repair of different DNA damage.

### 2.1 Base excision repair (BER) and nucleotide excision repair (NER)

The genome of all organisms are continuously experiencing subtle changes due to various genotoxicants generated endogenously such as reactive oxygen species (ROS), or environmental insults such as ionizing radiation and alkylating agents. The majority of these subtle changes in DNA such as SSBs are repaired through the base excision repair (BER) pathway. BER is initiated with damaged bases, which are then excised and replaced with newly synthesized DNA.^6^ In the next step, the apurinic/apyrimidinic (AP)‐endonuclease (APE) cleaves the AP site to form 3′ OH terminus at the damage site.^7^ Finally, the DNA polymerase and DNA ligase are recruited at the nucleotide gap produced by lesion base removal, thereby sealing the nick. Whereas BER is responsible for the repair of small lesions, the nucleotide excision repair (NER) is needed for bulkier SSBs that deform the DNA helical structure.^8^ The NER machinery involves a crucial protein, the excision repair cross‐complementing protein 1 (ERCC1), which takes an active part in the excision of DNA surrounding the lesion followed by replacement with normal DNA replication.^9^

### 2.2 Homologous recombination (HR) and nonhomologous end joining (NHEJ)

In mammalian cells, HR and NHEJ represent the two major pathways for repairing DSBs.^10^, ^11^ Since a homologous sister chromatid is required as a template for new DNA synthesis, HR pathways arguably repair DSBs during the S/G2 cell‐cycle phase, whereas NHEJ are active through all cell‐cycle phases except M phase. The HR analyses the homologous sequences from other parts of genome and thus collects the lost information at break sites. The HR pathway is initiated with the resection of break ends, followed by the formation of Rad51 nucleoprotein filament by Brca2 and Rad51, which retrieves the homologous sequence and promotes the formation of a joint molecule between the broken DNA and the homologous template.^12^ With minimal processing on DNA break ends, NHEJ is believed to be mechanistically simpler than HR, which directly rejoins the break ends together. The fundamental factor required for NHEJ is the heterodimer composed of Ku70/Ku80 and the catalytic subunit of the DNA‐dependent protein kinase (DNA‐PKcs) which recognize DSBs and facilitates downstream signaling factors for NHEJ, such as XRCC4, XLF, and DNA ligase IV.^13^ Although simpler among these repair mechanisms, NHEJ sometimes leads to rearrangements, especially the slow resection‐dependent NHEJ process, whereas HR is believed to be error free. However, in some cases, cross‐overs are formed in HR pathways, resulting in potential chromosomal rearrangements.^10^, ^14^, ^15^ These scenarios contribute to the preference of cells to NHEJ over HR in the absence of sister chromatid.

In addition to HR and NHEJ, a group of DSB repair pathways that share similar mechanisms to the two major DSB repair pathways, but are genetically distinct, are collectively known as alternative end‐joining (a‐EJ) pathways. The a‐EJ pathway can either share similar initiation process or constitute factors with HR,^16^, ^17^ but also with NHEJ in terms of DNA ends joining without homologous templates. Growing body of literature has reported that a‐EJ can cause gene deletions, translocations, and rearrangements in cancer cells.^18^, ^19^ Growing interest has been attached to a‐EJ pathways as potential therapeutic targets in cancer cells with compromised NHEJ or HR activities.^20^, ^21^, ^22^

### 2.3 Mismatch repair (MMR)

Apart from those produced by cells exposed to genotoxins, DNA damage can also derive from aberrant DNA processing. A DNA repair pathway targeting replication‐associated errors is known as MMR. During DNA synthesis, MMR corrects nucleotide misincorporation and thereby prevents permanent DNA change in dividing cells.^23^, ^24^, ^25^ Thus, defects in MMR either by gene mutation or epigenetic silencing may contribute to increased incidence of spontaneous mutation, which is typically associated with inherited and sporadic cancers.^26^, ^27^

### 2.4 Translesion synthesis and template switching

As an essential bypass mechanism for the repair of replication‐stalling DNA lesions, DNA damage tolerance (DDT) allows DNA replication across the obstructing element.^28^ The translesion synthesis (TLS) is one of the two distinct DDT modes that depends on the function of a special TLS polymerase, rather than replicative DNA polymerases, and directly replicates across the lesions.^29^ The TLS mechanism has been characterized as error‐prone due to the deficient proofreading activity of the TLS polymerase, which increases the risk of mutation. Not surprisingly, TLS is a major source of cellular mutagenesis.^30^ In contrast, another mode of DDT, the template switching (TS), involves recombination to a homologous DNA template on a sister chromatid, which is similar to the HR process and is believed to be more accurate in the outcome than TLS 21539841. The repair activities of TLS and TS start behind the replication fork, suggesting that they could occur during or after DNA replication, with TS beginning earlier in the S cell‐cycle phase and TLS in the late S phase.^31^, ^32^, ^33^

### 2.5 The Fanconi anemia (FA) pathway

Fanconi anemia is a rare genetic disease resulting from biallelic mutations of FANC genes, and affected patients are companied by deficient response to DNA damage.^34^, ^35^, ^36^, ^37^, ^38^ Affected patients have deficient ICL repair. The Fanconi anemia (FA) has been identified as a DNA repair pathway for its removal of a barrier that impedes DNA replication and transcription, the DNA interstrand crosslink (ICL).^39^ ICLs can be formed by aldehydes during multiple metabolic reactions such as lipid peroxidation and alcohol metabolism, and chemotherapies such as platinum.^40^, ^41^ Whereas intrastrand crosslinks are repaired by NER pathway as described above,^42^, ^43^ the highly toxic ICL is primarily repaired by the FA pathway.^44^ Following the detection of ICL by UHRF1 protein and the FANCM–MHF1–MHF2 complex, the FA core complex is recruited to chromatin and monoubiquitylates FANCD2‐I incorporation with UBE2T/FANCT E2 conjugating enzyme. Ubiquitylated FANCD2‐I recruits scaffolding protein for various DNA endonucleases, which split the strands near the ICL and facilitate the production of ICL‐derived double‐strand breaks. Given the considerable role that the FA pathway plays in DNA repair, it is not surprising that the FA pathway is also extensively studied in the context of cancer and that targeting the FA pathway is a potential cancer intervention strategy.^45^, ^46^

### 2.6 O^6^‐methylguanine‐DNA methyltransferase pathway

DNA methylating agents are known for their ability to inhibit DNA methylation and produce a wide range of DNA adducts, such as O^6^‐methylguanine (O^6^MeG) and O^4^‐methylthymine, which may result in base mispairing and subsequent point mutations.^47^ Given the smaller incidence of O^4^‐methylthymine production by methylating agents (< 0.3% compared with 8% of O^6^MeG),^48^ O^6^MeG is referred to as major source of methylating agents‐induced DNA adducts that cause mutagenesis and carcinogenesis.  O^6^MeG can be repaired by O^6^‐methylguanine‐DNA methyltransferase, also known as MGMT, in a single‐step suicide reaction.^49^ MGMT transfers the methyl at O^6^ site of damaged guanine to its cysteine residues, and thus prevents gene mutation. It is conceivable that MGMT reduces the efficacy of alkylating agents in cancer cells, potentially contributing to chemoresistance. Because DNA methylation can inhibit transcription, the methylation of MGMT promoter, which hampers its transcription, could be used to increase cell sensitivity to alkylating agents.^50^ A wide breadth of recent literature has identified the methylation of MGMT promoter as a response predictor for alkylating agents in gliomas.^51^, ^52^, ^53^, ^54^, ^55^

## 3 MECHANISMS UNDERLYING THE THERAPEUTIC APPLICATION OF DDR

As DNA‐damaging chemotherapies and ionizing radiation are used as the backbone of many therapeutic regimens in cancer, it is intriguing to speculate whether DNA repair deficiency represents a good source of anticancer therapeutic targets. Moreover, in some cases, the DDR deficiency is characterized as predicting biomarkers both for prognosis and treatment responses. A typical example has been discussed earlier in the review that MGMT promoter methylation can be used to predict the response to temozolomide in glioblastoma multiforme.^52^, ^56^ The underlying mechanisms for increased sensitivity of tumor cells to DNA‐damaging agents relative to normal cells lie in the three differentiating aspects: loss of at least one DDR pathways, elevated replication stress, and increased endogenous DNA damage.

### 3.1 DDR defects

Although DDR defects are implicated in the initiation and progression of cancers,^57^ defects in DDR pathways also provide therapeutic opportunities to target tumor cells with minimum impact on normal cells.^58^ Tumor cells carrying DDR deficiency leads to enhanced genomic instability and its dependency on remaining DDR pathways for survival. The combinational targeting of the remaining DNA repair pathways as a therapeutic approach reflects a concept known as synthetic lethality.^59^ The concept of synthetic lethality was based on two concurrent loss‐of‐function genetic events, either of which alone does not cause lethality but collectively contribute to cell death.^60^ As one genetic alteration on DDR pathways that are unique to cancer cells occurs, the second loss‐of‐function event caused by pharmacological inhibition with DDR inhibitor then becomes synthetic lethal to a cancer cells without affecting normal cells.^58^, ^61^, ^62^, ^63^

DNA‐damaging agents such as chemotherapies and radiotherapies have been used for years as the keystone of many anticancer therapeutics. Although these agents have demonstrated potent activity in a wide range of cancers, treatment resistance occurs through a variety of mechanisms and presents ongoing challenges including the upregulation of DDR components.^64^ DDR inhibitors were first developed as a combination partner for with platinum compounds, but later presented difficulty in application due to overlapping toxicities.^65^ Targeting DDR components as monotherapies is largely based on the concept of synthetic lethality.^66^ This approach would deliver considerable benefit to cancer patients compared with conventional treatments such as cytotoxic chemotherapies. Small‐molecule inhibitors targeting DDR are often DDR components that demonstrate enzymatic activities, including the PIKK family kinases, ChK1/2 and PARP‐1.

### 3.2 Replication stress

The intricate DNA replication system of Eukaryotic cells is tightly regulated during cell division by various proteins in cell cycles.^66^, ^67^ This is issue is a particularly prominent in the early S‐phase due to the fact that replication stress can be induced by untimely entry into S cell‐cycle phase before necessary molecules required for replication are generated.^68^ Numerous DNA nucleotides need to be accurately polymerized to ensure cellular homeostasis. Endogenous or exogenous obstacles that retard or terminate the progression of replication forks activate conserved cellular response pathways, which is referred to as replication stress. The molecular mechanism for replication stress is the stalled progression of DNA polymerase and the subsequent uncoupling of DNA polymerization from DNA helicases.^69^ One example of replication stress inducers are deficient G1/S cell‐cycle checkpoints, either caused by the loss of retinoblastoma tumor suppressor (pRb) function, deletion of the CDKN2A,^70^ or amplification of Cyclin D1 or Cyclin E.^71^, ^72^

Early stages of tumorigenesis is characterized with chronic replication stress and the subsequent collision of replication forks.^73^, ^74^ Some of collapsed replication forks are resolved by DDR pathways such as HR^75^ or mitotic DNA synthesis.^76^ However, increased genomic instability and mutagenesis can not be rescued in regions where the DNA replication process is not resumed. In order to accomplish bulk genome replication, cells often recruit error‐prone DNA polymerases. On the other hand, the replication failures and the subsequent presence of incompletely‐replicated DNA in mitosis would further lead to chromosomal entanglements between sister chromatids^77^ or the generation of micronuclei.^78^ Finally, if replication stress is not eliminated after mitosis, nuclear bodies, characterized by the DNA damage response protein p53 binding protein 1 (53BP1), are formed in daughter cells as protective machinery.^79^ Recent evidence has revealed an important role of RNA in DDR, particularly in human cells. Two substes of RNA were identified, damage‐induced long noncoding RNAs (dilncRNAs) and small DDR RNAs (DDRNAs).^80^, ^81^ The dilncRNAs potentially forms DNA–RNA hybrids and attracts DNA repair‐associated proteins such as BRCA1, BRCA2, RAD51, and MRE11 to the DNA damage sites and thus promotes DNA repair.^82^

Apart from being a crucial etiologic factor for cancer,^71^, ^83^, ^84^ elevated replication stress has also been observed during cancer therapies. Nucleoside analogues are widely used as chemotherapies such as acute myeloid leukemia (AML) induction therapy, which decrease the amount of dNTPs and delay DNA synthesis, and thus promote replication stress. For example, fluorouracil (5‐FU) is a pyrimidine analogue, which is incorporated into RNA following its conversion to 5‐fluoro‐deoxyuridine monophosphate (5‐FdUMP).^85^ In addition to RNA metabolism, 5‐FU has also been found to hamper DNA metabolism according to reported genetic screening results, which suggested increased 5‐FU sensitivity in cells deficient in the ATR‐Chk1 signaling pathway and homologous recombinational repair.^86^ Oxaliplatin, a platinum‐type chemotherapeutic drugs, inhibits DNA replication and G2/M cell‐cycle progression independent of ATM and ATR.^87^, ^88^ The underlying mechanism for the independence of oxaliplatin on DDR pathway lies in its ability to induce ribosome biogenesis stress by suppressing the transcription of deoxyuridine triphosphatase and the enzymes required for thymidylate biosynthesis.^89^, ^90^ Similar inhibitory effect on DNA synthesis can also be observed on TFTD (TAS‐102), a novel anticancer drug that suppresses dTTP biosynthesis^91^ and accelerates its incorporation into DNA.^92^

## 4 INHIBITORS TARGETING DNA REPAIR PATHWAYS

The current anticancer strategies that exploit DDR defects have largely been addressed by the development of targeted agents that inhibit molecules involved in DNA repair process. We herein summarized single‐agent DDR inhibitors currently under clinical trial development (Table 1).

**TABLE 1:** Single‐agent DDR inhibitors currently under clinical trial development

Target	Conditions	Interventions	Phase	Clinical trial*
PARP				
	Metastatic breast cancer	Drug: PARP inhibitor 2X‐121	Phase II	NCT03562832
	Breast cancer	Talazoparib	Phase II	NCT03990896
	Ovarian cancer	AK112	Phase I/II	NCT04999605
	Breast cancer	Rucaparib	Phase I	NCT03911453
	BRCA‐positive advanced breast cancer	KU‐0059436 (AZD2281)	Phase II	NCT00494234
	Ovarian cancer	EP0057 olaparib	Phase II	NCT04669002
	Pancreatic cancer	Niraparib	Phase II	NCT03601923
	Neoplasms	Talazoparib	Phase I	NCT03343054
	Ovarian carcinoma, breast cancer	AZD2281	Phase II	NCT00679783
	Advanced breast cancer	Talazoparib tosylate	Phase II	NCT02401347
	Advanced malignant solid neoplasm	Talazoparib	Phase II	NCT04550494
	HRR mutated solid tumors (VASTUS)	IDX‐1197	Phase I/II	NCT04174716
	Ovarian cancer	Niraparib	Phase II	NCT02354586
	Advanced tumors with ATM/BRCA1/2 gene mutation	Talazoparib	Phase II	NCT02286687
	Ovarian neoplasms	Niraparib	Phase III	NCT01847274
	Advanced/metastatic solid tumors	NMS‐03305293	Phase I	NCT04182516
	Solid tumor	RP12146	Phase I	NCT05002868
	Platinum sensitive BRCAm Serous ovarian cancer	Olaparib, Cediranib,AZD2281	Phase I	NCT02855697
	Ovarian neoplasms	KU‐0059436 (AZD2281)	Phase I	NCT00516373
	Ovarian cancer (neoadjuvant setting)	Niraparib	Phase II	NCT04284852
	Advanced tumors with HRR gene mutations	Olaparib oral capsule	Phase II	NCT03967938
	Ovarian cancer	Fluzoparib capsules	Phase III	NCT03863860
	Advanced malignant solid neoplasm	Olaparib	Phase II	NCT03212274
	Ovarian cancer	IMP4927	Phase III	NCT04169997
	Ovarian cancer	ZL‐2306 (nirapairb)	Phase III	NCT03709316
	Ovarian, breast cancer	Lynparza (olaparib)	Phase I	NCT04041128
	Ovarian cancer	ZL‐2306 (niraparib)	Phase II	NCT04392102
	Ovarian cancer	Talazoparib oral capsule	Phase I	NCT04598321
	Digestive cancers	Individualized PARP inhibitor	Not applicable	NCT04584008
	gBRCA mutated pancreatic cancer	Olaparib	Phase III	NCT02184195
	BRCAm pancreatic cancer	Olaparib	Phase II	NCT04858334
	Pancreatic cancer	RUCAPARIB	Phase II	NCT03140670
	Metastatic breast cancer	Olaparib		
	Relapsed ovarian cancer	Olaparib tablets	Phase III	NCT03534453
	Metastatic bladder urothelial carcinoma	Olaparib	Phase II	NCT03375307
	Advanced solid tumors	TALZENNA capsule	Phase I	NCT04672460
	Relapsed ovarian cancer	Olaparib tablets	Phase III	NCT01874353
	Stage IV pancreatic cancer	Olaparib	Phase II	NCT02677038
	HER2‐negative, germline BRCA mutation‐positive breast cancer	Niraparib	Phase III	NCT01905592
	Ovarian, fallopian tube, primary peritoneal cancer	Niraparib	Phase II	NCT03891576
	Metastatic castration‐resistant prostate cancer	Rucaparib	Phase III	NCT02975934
	Ovarian, fallopian tube, primary peritoneal cancer	Rucaparib	Phase III	NCT01968213
	Ovarian, fallopian tube, primary peritoneal cancer	Rucaparib		NCT04539327
	Prostatic neoplasms	Niraparib	Phase II	NCT02854436
	Breast cancer patients with chest wall recurrences	Olaparib	Phase I	NCT03955640
	gBRCAm breast cancer	Olaparib	Phase III	NCT02000622
	Biliary tract cancer with aberrant DNA repair gene mutations	Olaparib	Phase II	NCT04042831
	Solid tumors and with deleterious mutations in HRR genes	Rucaparib	Phase II	NCT04171700
	Ovarian, fallopian tube, or primary peritoneal cancer	Oral rucaparib	Phase II	NCT01891344
	Advanced malignant solid neoplasm	Olaparib	Phase II	NCT03233204
	Castration‐resistant prostate carcinoma	Olaparib	Phase II	NCT03516812
	Advanced malignant neoplasm	AMXI‐5001	Phase I/II	NCT04503265
	Metastatic carcinoma of the cervix	Nirapaib	Phase I/II	NCT03644342
	Solid tumor, adult	RBN‐2397	Phase I	NCT04053673
	Recurrent solid tumor	Olaparib	Phase II	NCT01078662
	Prostate, ovarian cancer	Rucaparib	Phase III	NCT04676334
	IDH1/2‐mutant Grade I–IV gliomas	Drug: PARP Inhibitor BGB‐290	Phase I	NCT03749187
	Advanced gastric adenocarcinoma	Olaparib	Phase II	NCT04209686
	Malignant mesothelioma	Rucaparib	Phase II	NCT03654833
	Acute myeloid leukemia	Olaparib	Phase II	NCT03953898
	Advanced or inoperable gastric cancer	Pamiparib (BGB‐290)	Phase II	NCT03427814
	Endometrial serous carcinoma	Niraparib	Phase II	NCT04716686
	Small cell lung carcinoma	IDX‐1197	Phase II	NCT03672773
	Urothelial carcinoma	Olaparib+EP0057	Phase I/II	NCT02769962
	Neoplasms	Niraparib tablet/capsule	Phase I	NCT03329001
	Advanced ovarian cancer	Olaparib tablets	Phase III	NCT01844986
	Head and neck squamous cell carcinoma	Niraparib	Phase II	NCT04681469
	Advanced solid tumors	JPI‐547	Phase I	NCT04335604
	Metastatic melanoma with HR mutation	Niraparib	Phase II	NCT03925350
ATM				
	Advanced solid tumors	M4076	Phase I	NCT04882917
	Neoplasms	BAY1895344	Phase I	NCT03188965
	NSCLC	VX‐970 (M6620)	Phase I/II	NCT02487095
	Cancers of the stomach and intestines	BAY 1895344	Phase I	NCT04535401
	SCLC, neuroendocrine cancer, pancreatic cancer	BAY 1895344	Phase I	NCT04514497
	Urothelial cancer	BAY 1895344	Phase I	NCT04491942
	Advanced cancers	LY2606368 (Prexasertib)	Phase II	NCT02873975
	Unresectable solid tumors	M1774	Phase I	NCT04170153
	Advanced stage solid tumors	M6620	Phase I	NCT03309150
ATR				
	Advanced solid tumor	RP‐3500	Phase I/II	NCT04497116
	Advanced solid tumors and lymphomas	BAY1895344	Phase I	NCT03188965
	Cancers of the stomach and intestines	BAY 1895344	Phase I	NCT04535401
	Advanced cancer	ART0380	Phase I/II	NCT04657068
	Unresectable solid tumors	M1774	Phase I	NCT04170153
	Pancreatic and ovarian cancer	BAY 1895344	Phase I	NCT04616534
CHK1				
	Advanced cancers	LY2606368	Phase II	NCT02873975
WEE1				
	Advanced solid tumors	IMP7068	Phase I	NCT04768868
	Uterine cancer	AZD1775	Phase II	NCT03668340
	Prostate cancer	Adavosertib	Phase II	NCT03385655
DNA‐PK				
	Advanced solid tumors, non‐Hodgkin's lymphoma, or multiple myeloma	CC‐122	Phase I	NCT01421524

* Data from https://clinicaltrials.gov.

### 4.1 Poly (ADP‐ribose) polymerase (PARP)

#### 4.1.1 Mechanisms underlying the application of PARP inhibitors

The development of PARP inhibitors represents the paradigm of the concept discussed earlier, known as synthetic lethality.^93^ PARP1 and PARP2 are key DDR enzymes that sense DNA damage and pass on signals by modifying target proteins with negatively charged poly(ADP‐ribose) (PAR) chains, known as PARylation.^94^ The structural changes of PARP1 following its binding to damaged DNA activate its catalytic function,^95^, ^96^ which facilitates the recruitment of DNA repair effector molecules and the structural remodeling of chromatins around DNA damage sites. In this way, PARP1 PARylates itself, a process known as autoPARylation, which potentially contributes to its release from repaired DNA.^97^ Recent advances in epigenetics have revealed the correlation of specific chromatin remodeling factors with DDR.^98^ One such example is PARP1, which PARylates MORC2 and increases its ability to induce chromatin remodeling. Since eukaryotic DNA is surrounded by condensed chromatin, the dynamic remodeling of chromatin would largely affect the efficiency of DNA repair.^99^, ^100^ More studies are thus warranted to shed light on the collaborative interplay between chromatin‐associated enzymes and DDR. Given the pivotal role of PARP in promoting the effective repair of DNA, PARP inhibitors selectively kill tumor cells with homologous recombination deficiency. Conflicting results were reported regarding whether PARP is required for BER,^101^ with some evidence suggesting the increased sensitivity of PARP1‐deficient cells to base‐damaging agents,^102^, ^103^, ^104^ whereas some studies found that PARP was not necessary for the repair of base.^105^

Alongside the inhibition on enzymatic activities of PARP, the process referred to as PARP trapping provides an additional mechanism for PARP inhibitors, where PARP1 and PARP2 are trapped at the site of DNA damage and block the recruitment of proteins involved in DNA repair. Since a complete set of repair‐associated proteins is the prerequisite for accurate DNA repair, PARP‐inhibited cells lost the capacity to properly repair their DNA during replication, eventually inducing mitotic catastrophe and subsequent cell death.^94^ Multiple PARP inhibitors have demonstrated comparable antitumor efficacy and selective inhibition on PARP1 and PARP2, but their abilities to induce PARP trapping vary, which contributes to the difference of recommended doses among PARP inhibitors.^106^, ^107^

PARPi is a promising therapeutic strategy for BRCA‐mutant tumors, which is a typical setting of synthetic lethality.^108^ BRCA gene has long been identified as crucial components of the HR pathway.^109^ In cells harboring BRCA mutation, alternate DNA repair mechanisms such as the PARP pathway are initiated to fix the damage. Thus, PARP inhibition in a BRCA‐deficient setting likely causes the accumulation of DNA damage and thereby leads to cell death. However, as cells with BRCA1 or BRCA2 germline mutation are unable to fix treatment‐induced DSBs, toxicity caused by PARP inhibitor has received considerable attention. Previous studies investigated the association between myelosuppression occurrence and BRCA1 or BRCA2 mutation status in patients receiving platinum‐based chemotherapy and revealed no significant correlation between BRCA mutation status and hematological toxicities.^110^ However, it remains unclear whether PARPi toxicity could also be used as a predictive biomarker for PARPi treatment response.

#### 4.1.2 PARP inhibitors as the first‐line therapy

Ovarian cancer is the leading cause of gynecologic cancer‐related deaths in women worldwide,^111^ and the standard care for the newly diagnosed advanced ovarian cancer (NADOC) patients in the last two decades is the surgical debulking followed by platinum–taxanes‐based systemic chemotherapy. Unfortunately, an estimated number of 70% of patients with advanced ovarian cancer experience relapsed disease within 3 years posttreatment.^112^ The concurrent and maintenance anti‐VEGF bevacizumab was later recommended for the standard first‐line systemic treatment of epithelial ovarian cancer, which improves PFS in patients with higher risk of recurrence (International Federation of Gynecology and Obstetrics FIGO stage IV or suboptimally debulked stage III ovarian cancer—OC).^113^ However, the efficacy of the combinational treatment diminishes over time with a 5‐year survival rate being around 35%, and adverse effects accumulate as chemotherapy cycles proceed.^114^, ^115^, ^116^ Thus, recent research of this field aims to identify more efficient drug combinations to aid the systemic treatment of ovarian cancer patients.

**FIGURE 2:**
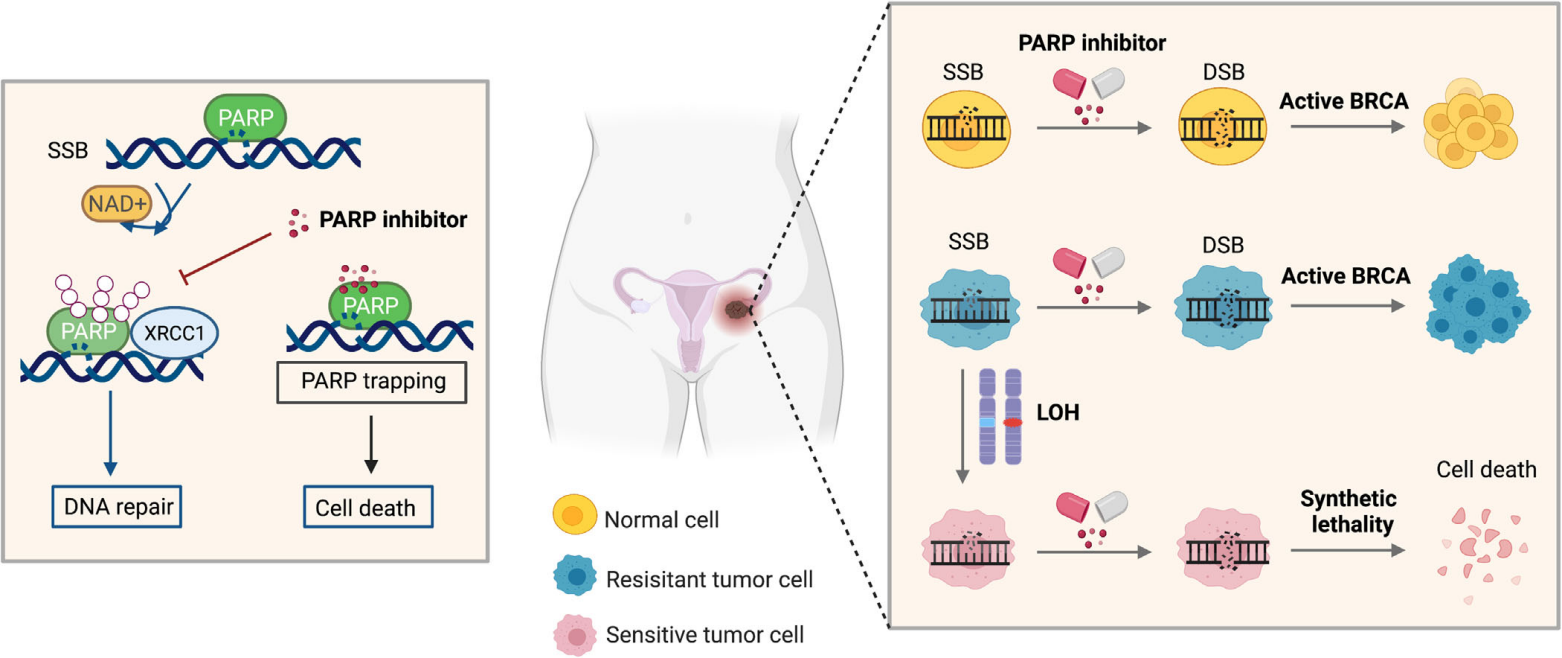
The mechanism underlying the application of PARP inhibitors in patients with advanced ovarian cancer. SSBs, single‐strand breaks; DSBs, double‐strand breaks. Figure was created with Biorender

In a recent European Society for Medical Oncology (ESMO) Congress, research teams reported preliminary results from clinical trials of three different PARP inhibitors in patients with ovarian cancer, including the PAOLA‐1/ENGOT‐OV25 Phase III trial where the combination of PARP‐inhibitor olaparib and bevacizumab was assessed for the first time as maintenance therapy following platinum‐based chemotherapy in the overall population regardless of the BRCA status.^114^, ^115^, ^116^ The mechanism underlying the application of PARP inhibitors in patients with advanced ovarian cancer is illustrated in Figure 2. Following the promising results from these trials, the oncology community starts to review the practice regime of PARP inhibitors in first‐line treatment of NADOC and the selection criterion for patients that would receive the maximum benefits. The defined subset of patients based on their molecular diagnosis include those with BRCA‐mutation, HR‐deficiency, and HR‐proficiency.^117^ Here, we discuss the updated data from the ongoing as well as previous clinical trials regarding the application of PARP inhibitors.

##### Olaparib

The first human clinical trials of PARPi evaluated the chemopotentiation effect of low‐dose rucaparib in patients with metastatic melanoma.^118^ Currently, four PARP inhibitors, olaparib, rucaparib, niraparib, and talazoparib, have been approved by the US Food and Drug Administration (FDA). Based on accumulating research results on synthetic lethality observed between PARP inhibition and BRCA mutation status,^119^, ^120^ a clinical evaluation of olaparib was initiated in 2005, where 63% of patients cancer with germline BRCA1 or BRCA2 mutations (gBRCAm) exhibited durable clinical benefit.^121^ The evaluation of olaparib later extended to patients with gynecological malignancies and reported a favorable response to olaparib in patients who response better to prior platinum chemotherapies. This finding accorded with the hypothesis that platinum‐based therapies and PARPi shared similar molecular targets.^122^ Phase II trials further supported significant clinical benefit in multiple gBRCAm cancer types including breast, ovarian, pancreatic, or prostate cancers.^123^, ^124^, ^125^ In 2014, olaparib was approved as maintenance therapy for platinum‐sensitive advanced ovarian cancer with germline BRCA1 or BRCA2 mutations (gBRCAm).^126^ More recently, a randomized Phase III trial reported improved survival outcomes in gBRCAm/HER2‐negative breast cancer patients receiving olaparib than those with standard chemotherapy.^127^

A growing number of clinical trials have been conducted since 2009 to investigate the efficacy and safety of PARP inhibitors in multiple cancer types irrespective of the BRCA status.^128^, ^129^, ^130^, ^131^, ^132^ A Phase II trial metastatic investigated the treatment response to olaparib in patients with castrate‐resistant prostate cancer (mCRPC) by evaluating clinical parameters including PSA decline and radiologic responses.^133^ Notably, the overall response rate in unselected CRPC population to PARP inhibitors was only 33%, possibly attributed to the observed tumor mutations in other DDR members.^134^ The team then conducted next‐generation sequencing on enrolled patients and the genetic map of these patients revealed homozygous deletions or mutations in DRR‐associated genes including ATM, PALB2, CHEK2, FANCA, and HDAC2. This trial not only granted olaparib approval for the treatment of BRCA1/2‐ or ATM‐mutant mCRPC patients, but also provided additional application of PARPi in DDR‐defective patients beyond BRCA mutations. Thus, it may be insufficient only to use BRCA1 or BRCA2 mutations as predictive biomarker for PARPi responders. Based on the observation that ATM gene alteration resulted in increased sensitivity of cells to PARP inhibition, ATM gene mutation was included as a predictive biomarker for PARPi response in the FDA breakthrough therapy designation.^135^, ^136^ It has to be addressed that the ideal predicting factor for PARPi response would be recombination deficiency, which does not exist in practice.

##### Rucaparib

The combination of rucaparib and temozolomide were the first clinical trial containing PARPi treatment regimens.^118^ Rucaparib was first indicated for the treatment of advanced ovarian cancer with either germline or somatic BRCA1/2 mutations, and was then approved in 2018 for the maintenance treatment of platinum‐sensitive ovarian, fallopian tubal, and peritoneal cancer regardless of the BRCA status.^137^ In the maintenance setting (ARIEL 2, NCT01891344), advanced ovarian cancer patients were divided into three groups based on the genomic features of their tumors including the germline or somatic BRCA  status and chromosomal loss of heterozygosity (LOH). The longest progression‐free survival (PFS) was observed in the BRCA mutant group, followed by the high LOH group.^138^ BRCA status appeared to be a significant predictor in the maintenance setting of rucaparib, given that the proportion of BRCA wild‐type patients displaying durable responses was smaller than that of patients receiving standard platinum‐based chemotherapies.^139^ Thus, the following Phase III trial (NCT01968213) aimed to investigate the potential of the genome‐wide LOH to be transformed into a clinically applicable biomarker for patients’ responses to rucaparib 27908593. Along with the promising results from an additional Phase II trial HGSOvCa (NCT01482715),^140^ rucaparib was approved for chemotherapy‐pretreated patients with gBRCAm or sBRCAm advanced ovarian cancer. However, rucaparib has been reported as the least selective clinical PARP1 inhibitor with simultaneous inhibition on multiple PARPs ranging from PARP1, PARP2 to mono(ADP‐ribosyl) transferases PARP3, PARP4, PARP10, PARP15, and PARP16.^141^, ^142^

##### Veliparib and niraparib

Some PARP1/2 inhibitors are not highly selective such as rucaparib discussed earlier.^142^ For example, niraparib has been reported to interact with non‐PARP targets such as deoxycytidine kinase (DCK).^143^ The cross‐inhibition on DCK, which is fundamental for the activation of nucleoside analogs, would decrease the efficacy of niraparib/gemcitabine synergy.^143^ On the other hand, due to its formation of a PARP1/2‐unique water‐mediated hydrogen bond that interacts with a highly conservative subdomain D766, veliparib has been identified as the most selective clinical inhibitors targeting PARP1/2, with 100‐fold higher affinities to PARP1/2 relative to olaparib and talazoparib.^144^ In a Phase III clinical trial, the median duration of PFS was significantly increased in ovarian cancer patients receiving niraparib, irrespective of gBRCA status (NCT01847274).^145^ Though non‐gBRCA mutant, these tumors were identified with a unique mutational profile similar to the genome of gBRCAm tumors, which is referred to as BRCAness DNA scar.^146^ Though BRCAness DNA‐scar positive patients appeared to have improved prognosis compared to BRCAness‐scar negative patients, the prognostic value of BRCAness‐scar as a predictive biomarker remains incompletely defined and requires further clarification in larger cohorts.^139^, ^145^

Though effective in the clinical practice, PARP inhibitors have also demonstrated certain limitations like any other novel development in history. Predominantly, the varying PARP trapping ability by different PARP inhibitors potentially lead to the off‐target PARP trapping on the DNA of normal cells.^147^ Besides, the emerging resistance to PARP inhibitors also poses challenges to their clinical application, the underlying mechanisms of which include loss of PARP trapping,^148^, ^149^ upregulated drug efflux protein expression,^150^, ^151^ stabilized replication fork stabilization,^152^, ^153^, ^154^ and the restoration of HR pathway.^155^, ^156^, ^157^, ^158^, ^159^, ^160^, ^161^, ^162^, ^163^

### 4.2 Poly(ADP‐ribose) glycohydrolase (PARG)

The above limitations of PARP inhibitors motivated the design of additional therapeutic targets for BRCA‐proficient and deficient tumors, or PARPi‐resistant tumors. PARG reverses the action of PARP enzymes by hydrolyzing the ribose–ribose bonds in PAR following DNA damage.^164^, ^165^, ^166^ Likewise, the active role of PARG in DNA replication and repair leads to increased sensitivity to DNA damaging agents in PARG‐deficient cells. Though extensive studies have suggested the correlation between PARP inhibitors and synthetic lethality, research on therapeutic mechanisms of PARG inhibition has lagged behind. It has been reported that depletion of the HR proteins such as BRCA1/2 in breast cancer cells could stimulate synthetic lethality in PARG‐inhibited cells,^167^, ^168^ and that COH34, a PARG inhibitor, is able to induce cell death of ovarian and breast cancers with BRCA mutations or resistance to olaparib.^169^ However, conflicting results were reported in other cancer cells.^170^ Of the six tested breast cancer lines, only one BRCA‐proficient cell line was sensitive to PARG inhibitor PDD00017273, whereas five cell lines failed to respond to PDD00017273 including those with BRCA mutations.^171^

PDD00017273 is a quinazolinedione‐type PARG inhibitor with improved specificity, efficiency, and cell permeability, but lacks bioavailability.^172^ Unlike cytotoxic PARP inhibitors, the major effect by PDD00017273 is cytostasis where the replication catastrophe does not progress into mitosis but rather remains static in interphase.^171^ However, the exposure to ionizing radiation enhanced centrosome amplification and the subsequent multipolar spindle formation and chromosome missegregation caused by PARG deficiency.^173^, ^174^ Thus, it is intriguing to speculate that under some circumstances such as PARG inhibition coupled with cell‐cycle checkpoint blockades or DNA‐damaging agents, mitotic abnormalities would occur.^175^, ^176^, ^177^

Neither of the first‐generation PARG inhibitor (GPI 16552 and gallotannin) demonstrates sufficient activity in vitro and its frequent off‐target effects in cells makes it a less than ideal strategy.^178^, ^179^ Another early PARG inhibitor, rhodanine‐based PARG inhibitor (RBPI) is more selective than previous generation PARGi, with limited cell permeability.^180^, ^181^ The recently reported COH34 is a novel small‐molecule PARG inhibitor with nanomolar potency both in vitro and in vivo, and notably, with efficiently killing effect on PARP inhibitor‐resistant cancer cells, which makes it a good candidate for clinical studies.^169^ Chemical library screening identified methylxanthine derivatives JA2–4 and JA2131 as selective bioavailable PARG inhibitors, which showed comparable killing on PARP inhibitor‐resistant A172 glioblastoma cells.^182^

### 4.3 Ataxia telangiectasia mutated (ATM)

The DDR signaling cascades are driven by serial protein phosphorylation. ATM, ATR, and DNA‐PKs are the key kinases involved in this process and are similar in molecular structure, the C‐terminus of which is responsible for phosphorylation activity especially on serine or threonine residue (Ser/Thr).^183^, ^184^, ^185^ Activated by DNA double‐strand breaks, ATM is recruited to DSB sites by the MRE11‐RAD50‐NBS1 (MRN) complex.^186^ Substrates of ATM include p53, CHK1, and CHK2, the phosphorylation of which would lead to intra‐S or G2/M cell‐cycle arrest.^187^, ^188^ Despite its canonical role in a wide variety of molecular processes such as DNA repair, ATM has also been characterized with noncanonical functions including spliceosome displacement.^189^ As ATM is rightly considered as a tumor suppressor, ATM deficiency or deleterious alterations are commonly seen in solid tumors and B‐cell lymphoma.^190^ Germline ATM mutation likely contributes to Ataxia Telangiectasia (A‐T), a neural degeneration disorder characterized by increased predisposition to cancer.^191^

The main reason for ATM deficiency in cancer cells is hypermethylation of the ATM promoter,^192^ with multiple cancer types including brain cancer, breast cancers, lung cancer, and head and neck squamous cell carcinoma exhibiting hypermethylated ATM promoter region.^193^, ^194^, ^195^, ^196^ However, ATM signaling can also be advantageous to tumors, increasing their risks of therapeutic resistance to radiation and chemotherapies.^197^ Several ATM inhibitors are now under investigation for cancer therapy.^198^, ^199^ The loss of ATM occurs in prostate cancer and was recently suggested to increase cell sensitivity to ATR inhibition.^200^

The first reported selective ATM inhibitor, 2‐morpholin‐4‐yl‐6‐thianthren‐1‐yl‐pyran‐4‐one, (KU‐55933), was developed by screening the PIKK family‐targeting compound library and exhibited 100‐fold higher selectivity for ATM over ATR, DNA‐PK, and PI3K.^199^, ^201^ Exposure to KU‐55933 sensitizes cells to cytotoxic agents that cause DSB, by blocking HR repair signals and thereby increasing γ‐H2AX and RAD51 foci accumulation.^202^ In response to chemotherapy, KU‐55933 inhibits ATM‐mediated repair signals in the presence of inositol polyphosphate‐4‐phosphatase type II (INPP4B), which has contradictory roles in cancer progression.^203^ In colon cancer cells, INPP4B acts as an oncogenic factor that positively regultates AKT 26411369, whereas INPP4B suppresses cancer progression in prostate cancer cells by reducing tumor migration, invasion, and angiogenesis.^204^

KU‐60019 is an analogue of KU‐55933 with improved pharmacokinetics and bioavailability and is reported to interrupt radiation‐induced ATM phosphorylation in glioma cells.^205^ Given that PTEN is an active participant of DNA repair process, it is not surprising that KU‐60019 was specifically toxic to PTEN mutant cancer cells.^206^ Besides, the combination of KU‐60019 and cisplatin would induce synthetic lethality in PTEN‐deficient cells,^207^, ^208^ the underlying mechanism of which involves increased PARP cleavage and γ‐H2AX formation.^209^ Thus, PTEN‐deficiency is a potential biomarker for predicting repines to DDR‐targeting agents. KU59403 is the first ATM inhibitor tested in preclinical trials with improved solubility, bioavailability, and selectivity.^210^ KU‐59403 potentiates the efficacy of chemotherapies and IR at low doses in cancer cells irrespective of TP53 mutation status.^210^ However, KU‐59403 monotherapy failed to demonstrate antitumor effects either in vitro or in vivo, which largely limited its clinical application and was not widely used thereafter. CP466722 was identified as a ATM kinase inhibitor by screening targeted compound library, which does not display inhibitory activities on PI3K family members. Noteworthy, even transient inhibition of ATM by CP466722 is sufficient to induce radiosensitization in cells and suggests that therapeutic radiosensitization, indicating that ATM is required for early stage of the DDR process.^211^

The major limitations of earlier developed ATM inhibitors are their bioavailability in central nervous system via the blood brain barrier (BBB). Modified ATM inhibitors AZ31 and AZ32 have higher free brain concentrations and their radiosensitization effects were more prominent in p53 mutant cells than p53 wild‐type glioma cells.^212^ In contrary to this finding, previous evidence suggested increased sensitivity of wild‐type p53 glioblastoma cells to radiation than p53 mutant cells.^213^ AZD0156 has been reported to enhance the efficacy of DSBs in mouse xenograft models but lack BBB penetration.^214^ The further optimized compound, AZD1390, is now under investigation as a radiosensitizer for nervous system malignancies.^215^

### 4.4 Ataxia telangiectasia and Rad3‐related protein (ATR)

In contrast to ATM, which is triggered by DSBs, ATR is activated by and recruited to replication protein A (RPA)‐coated single‐strand DNA (ssDNA).^216^, ^217^ Single‐strand DNA can be produced by nucleolytic processing of DSBs as well as the uncoupling of the replicative DNA helicase from the DNA polymerase machinery. The intracellular ATR signaling involves the phosphorylation of a series of downstream molecules, triggering a wide array of responses including blocking cell‐cycle checkpoints, DDR, and cell apoptosis.^218^ In response to genotoxic stress, Chk1 is phosphorylated on serines 317 (S317) and 345 (S345) by ATR, thereby activating WEE1,^219^, ^220^ which in turn phosphorylates CDK1 on tyrosine 15 and suppresses mitotic entry.^221^ CDC25A is responsible for removing the inactivated phosphates on CDK2. Once CDC25A is phosphorylated by CHK1, the activation of intra‐S phase checkpoints impairs the rate of CDC25‐mediated replication, allowing cells to repair DNA damage. In addiction, as CDK1 is fundamental for the progression via G2/M checkpoints, ATR has been described colloquially as the apex of DDR signaling that acts on both S and G2/M cell‐cycle checkpoints, preventing the entry of damaged DNA into replication process before it has been properly repaired.^187^, ^222^, ^223^, ^224^

Cancer‐associated inflammation and cytotoxic treatments such as chemotherapies and radiotherapies are known to cause replication stress, which increases cell reliance on the ATR‐mediated S and G2/M checkpoints activation as countermeasures. Thus, it is intriguing to speculate whether inhibition of ATR would sensitize cells to DNA damaging agents such as chemotherapy, encouraging the development of selective ATR inhibitors. However, compared with other DDR proteins such as PARP, development of ATR inhibitors has lagged behind. Contributing factors may include the large size of the ATR molecule and the lack of knowledge on its crystal structure. In addition, its highly homologous active sites in all PIKKs and the demand for coactivating proteins further restrict its drug design.

The first chemicals reported to inhibit ATR were natural molecules caffeine and schisandrin B, the inhibition of which was nonspecific and only worked at high concentrations.^225^, ^226^ This finding further confirmed the potential of natural compound for future synthesis of DDR‐regulating drugs.^227^ Several approaches were used to identify potentially potent ATR inhibitors. One such example is the cell‐based high‐throughput microscopy that enables the screening of compounds, investigating their specific activity on ATR,^228^, ^229^ where they identified a highly selective compound, ETP‐46464 with specific action on ATR, rather than ATM or DNA‐PKcs.^229^ Recent advancement in gene editing suggests that CRISPR DDR screens can also be used to identify drug candidates.^230^

Another identification strategy is the in vitro use of recombinant ATR to test its kinase reactions, through which researchers were able to characterize compounds that directly and specifically targeted ATR, such as VE‐82.^66^, ^231^ With further modification on pharmacological properties, VE‐821 was later named VE‐822 and is now under clinical investigation as VX‐970 (M6620) (NCT03309150, NCT03022409, NCT02723864, etc.).^232^ Interestingly, some ATR inhibitors were discovered during research on inhibitors developed for other targets. NU6027 was originally selected for CDK2 inhibition and was later found to impair HR pathway, thereby sensitizing cells to DNA‐damaging agents and PARP inhibitors.^233^ The new‐generation ATR inhibitors include AZD6738, an derivate of the compound AZ20, which is currently under clinical investigations (NCT02567422, NCT03022409, NCT02157792), BAY1895344 (NCT03188965),^234^, ^235^, ^236^ berzosertib (NCT02157792),^237^ and a recently reported pyrazolopyrimidine‐containing inhibitor of ATR.^238^

### 4.5 CHK1

As described earlier, CHK1 is actively involved in the ATR‐ and ATM‐initiated DNA damage response by phosphorylating and recruiting a series of regulatory proteins. CHK1 regulates the intra‐S checkpoint by phosphorylating CDC25A, leading to the degradation of CDC25A and the subsequent decrease of cyclin‐dependent kinase 2 (CDK2) activity in S cell‐cycle phase,^239^, ^240^ and the phosphorylation of CDC25C and WEE1 by CHK1 regulates mitotic entry and G_2_/M checkpoints.^241^ Moreover, CHK1 also phosphorylates RAD51 on Thr‐309 promoting its interaction with BRCA2 during HR.^242^, ^243^, ^244^, ^245^ Noteworthy, CHK1 also acts on a number of physiological processes that are critical to cell survival. For example, the suppression of CHK1 leads to p53‐induced death domain (PIDD) signaling and the associated caspase 2‐mediated cell death.^246^ It has been recently reported that the phosphorylation of nucleophosmin (NPM) by CHK1, a chaperone protein involved in various cellular functions including, disrupts its interaction with PIDD, thus protecting cells from caspase 2‐mediated cell death.^247^ Further studies are warranted to clarify the importance, yet poorly defined role, of CHK1 in other cellular processes independent of DDR.

Though CHK1 deficiency has been reported to induce early embryonic lethality in vivo,^219^ the knockdown of which is preferentially more deleterious in tumor cells than in normal cells, suggesting the potential of Chk1 as a therapeutic target in cancer treatments. On the other hand, increased CHK1 levels have been reported to correlate to worse prognosis, disease recurrence, and therapeutic resistance,^248^, ^249^, ^250^, ^251^, ^252^ further supporting the therapeutic potential of Chk1 inhibition. In circumstances where cells harbor certain genetic alterations, such as c‐MYC, CHK1 inhibitors are able to induce synthetic lethality in malignancies driven by oncogene c‐MYC.^253^, ^254^, ^255^ Likewise, CHK1 inhibitor PF‐00477736 exhibited cytotoxic effects on mantle cell lymphoma (MCL) and myeloma with translocation t(11;14)‐mediated Cyclin D1 overexpression.^256^, ^257^ Cells with acquired PF‐00477736‐resistant cells displayed enriched prosurvival and proliferation‐associated gene patterns, suggesting that inhibition of prosurvival signaling pathways could potentially sensitize cells to CHK1 inhibitors.

The first‐generation CHK1 inhibitors were used as chemosensitizing agents, the majority of which were nonspecific due to their high affinity to plasma protein 1‐acid glycoprotein, with a long half‐life and low bioavailability.^258^ The early CHK1 inhibitors were mostly used as combinational partners with cytotoxic agents in cancer,^187^, ^259^ the clinical development of which was largely restricted by their unacceptable toxicities and suboptimal pharmacological profiles.^260^ With significantly improved selectivity toward CHK1, the second‐generation CHK1 inhibitors such as LY2606368, LY2880070, SRA737, and GDC‐0575 are now under intense clinical studies. These CHK1‐targeting agents potently synergize with drugs that produce DNA damage including cytotoxic chemotherapies and antimetabolites.^261^, ^262^ One such example is the combinational treatment of low‐dose gemcitabine with GDC‐0575, which induced promising objective response rates in  patients with advanced sarcoma.^263^

Recently, clinical trials (NCT02797977, NCT02797964) reported promising results that the combination of a novel CHK1 inhibitor SRA737 with low‐dose gemcitabine led to partial responses in 6 patients and stable disease for at least 4 months in 32 patients. SRA737 has also demonstrated synergistic effect with PARP1 inhibitors in cancer both in vitro and in vivo.^264^ Despite intense interest in CHK1 inhibitors, no known agents have reached Phase III clinical trial or received FDA approval. According to preclinical results, though the single use of CHK1 inhibitors did not usually cause significant toxicities, the unacceptable cytotoxic effects on normal cells caused by the combination therapy with DNA damaging agents outweighed the modest gains.

### 4.6 WEE1

In response to DNA damage, the activated ATR phosphorylates Chk1, which in turn phosphorylates WEE1 and CDC25.^265^, ^266^, ^267^ In contrast to CDC25 whose activity is suppressed by the phosphorylation, WEE1 is activated and then phosphorylates downstream CDK1 on Tyr15 and Thr14 to inhibit its activity, leading to G2/M cycle arrest and allowing time for DNA damage repair. In addition, by phosphorylating CDK1 on Tyr15, WEE1 also prevents the progression of S phase to G2 phase before DNA replication is completed.^268^ Moreover, WEE1 has also been reported to phosphorylate histone H2B on Tyr37, thereby blocking the transcription of certain histone genes that reduce the burden of the histone mRNA turnover machinery.^269^

G1/S and G2/M checkpoints are regulated by p53 gene, which is frequently absent or deficient in cancer cells. Under this circumstance, cancer cells become highly dependent on WEE1‐mediated G2/M checkpoint control for DNA repair.^270^, ^271^ It is thus not surprising that some cancers are accompanied by WEE1 overexpression, which decreases their sensitivity to radiotherapy and chemotherapy.^272^, ^273^ Besides, results from whole‐genome characterization of chemoresistant ovarian cancer suggested the feasibility of WEE1 inhibition in multiple tumor‐related pathways.^21^, ^274^ These evidence support the early therapeutic rationale of WEE1 inhibitors in p53‐deficient tumors. It is becoming increasingly clear that neither p53 deletion nor the loss of G1 checkpoint is a predictor for WEE1 sensitivity.^275^, ^276^, ^277^ Currently, most clinical studies focus on the combinational use of WEE1 inhibition with chemotherapeutic drugs, which will be discussed further in the review.

The first generation of small‐molecule WEE1 inhibitors, represented by PD0166285, was rather unspecific with an inhibitory activity against multiple kinases such as EGFR, CHK1, and c‐Src.^278^, ^279^, ^280^ The first selective WEE1 inhibitor, adavosertib (AZD1775), was obtained from screening a small‐molecule compound library.^281^ Though more selective than previous‐generation WEE1 inhibitors, from kinase profiling results, AZD1775 was found to target other kinases as well with reduced potency.^282^, ^283^ For example, the unspecific targets of AZD1775 include PLK, the role of which in cell‐cycle progression has been described as antagonistic to WEE1. This multiple binding may contribute to the difficulty in interpreting experimental results, but it was recently suggested that therapeutic concentrations of AZD1775 were not sufficient to suppress PLK1 activities.^284^ Noteworthy, AZD1775 exhibits potent antitumor activity even as monotherapy.^285^ Given that single‐agent therapy is believed to be almost equally toxic to normal and cancer cells, the antitumor activity of WEE1 inhibitors monotherapy potentially arises from the increased replication stress in cancer cells.^286^, ^287^, ^288^

Whereas the rationale for WEE1 inhibitors is clear, its clinical application is restricted by its demand for appropriate therapeutic windows. The substantial >grade 3 adverse effects caused by AZD1775 are often a concern (NCT02341456, NCT02666950, NCT01357161, NCT00648648). As WEE1 is required for a number of physiological processes in normal cells, adverse events are usually expected to impact cells undergoing frequent divisions such as the hematopoietic system and intestinal epithelium.^289^ For this reason, numerous efforts have been undertaken to optimize dosing and therapeutic schedule of AZD1775,^290^ with its analogues being developed, which remained effective but brought lower toxicity.^291^ Another research attempt is to identify additional biomarkers for AZD1775 to reduce the off‐target effects. AZD1775 is able to induce synthetic lethality in cells with defects in the Fanconi Anemia or HR pathways,^285^, ^292^ suggesting that the efficacy of AZD1775 may be enhanced by further inhibiting additional factors that downregulate DNA replication.

### 4.7 DNA‐PK inhibitors

DNA‐dependent protein kinase was initially discovered by chance in 1985 when scientists added double‐stranded DNA (dsDNA) into the cell extracts and identified this protein with enhanced phosphorylation.^293^ Later in 1990,  the DNA‐dependent protein kinase catalytic subunit (DNA‐PKcs) was identified.^294^, ^295^ Encoded by the PRKDC/XRCC7 gene, DNA‐PKcs is abundantly present in human cells with no fewer than 50,000 molecules per cell and the largest PIKK family member.^296^, ^297^, ^298^, ^299^, ^300^ DNA‐PKcs shares similar domain compositions with two other PIKK family members involved in DDR, ATM, and ATR, such as the kinase domain and the conserved FRAP‐ATM‐TRRAP (FAT) domain.^301^

Loss of the key factors in the NHEJ pathway has long been considered as a hallmark for tumor progression and increased sensitivity to DSB‐inducing agents, possibly due to increased genomic instability.^298^, ^302^, ^303^ The upregulation of DNA‐PK expression was observed in various tumor types including the gastrointestinal cancer, lung cancer, and hepatocellular carcinoma and was associated with higher tumor grades and poor prognosis.^304^, ^305^, ^306^ In melanoma, increased DNA‐PKcs expression was related to a progressed phenotype with tumor microenvironment favoring metastasis.^307^ In addition, DNA‐PKcs upregulation has been reported to promote resistance to radiotherapy and chemotherapy in thyroid,^308^ nasopharynx,^309^ cervix cancers,^310^ and leukemia.^310^, ^311^ Moreover, DNA‐PK has been reported to transcriptionally regulate protumorigenic pathways, leading to tumor progression and metastasis.^312^, ^313^These findings have encouraged the design of multiple DNA‐PK inhibitory strategies.

Giving the structural similarity between DNA‐PK and PI3K, early attempts to block DNA‐PK were based on pharmacological approaches that directly targeted PI3K or its derivatives. Development of DNA‐PK inhibitors mainly focuses on the catalytic activity of DNA‐PKcs, whereas novel anti‐DNA‐PK approaches such as DNA‐PKcs‐inhibiting microRNAs^314^, ^315^ or inhibitors targeting the Ku heterodimers were based on the homology model of the ATP‐binding site.^316^, ^317^ The first reported DNA‐PK inhibiting compound was caffeine, which was identified with in vitro kinase activities on two other DDR master kinases ATM and ATR, and later with inhibition on DNA‐PK.^318^ Further application of these early DNA‐PK inhibitors such as wortmannin^226^ and vanillin^319^ was limited due to poor selectivity and complexed structure. With the advent of a lead compound LY294002, more specific and potent derivate compounds were later developed such as NU7441, NU7427, NU7026, and NU7163.^320^, ^321^, ^322^, ^323^

In preclinical studies, NU7427 and NU7026 potentiated the therapeutic effect of IR and topoisomerase II inhibitor chemotherapy in cancer cells,^321^, ^324^ whereas NU7441 substantially delayed the repair of IR‐ and chemotherapy‐induced DSBs both in vitro and in vivo.^325^ There were compelling preclinical data studies suggesting NU7441 as a potent DNA‐PK inhibitor in cancer models.^326^, ^327^, ^328^, ^329^, ^330^ Another class of DNA‐PK‐targeting compounds studied in preclinical studies are a series of arylmorpholine‐containing compounds derived from IC60211,^331^ which include IC86621, IC486154, IC87102, and the most intensively used IC87361.^332^, ^333^ Despite extensive research, clinical evaluation and application of these inhibitors could not be achieved due to their undesirable pharmacokinetics.^334^

VX‐984 and M3814 are the new‐generation DNA‐PK selective inhibitors, which have already progressed into clinical trials in combination with IR or chemotherapy. VX‐984 is known for its potential to cross the blood brain barrier based on the observation that VX‐984 enhanced the response to radiotherapy in glioblastoma mouse models.^335^ M3814 has been reported to suppress NHEJ repair induced by chemotherapies and radiation, and to enhance the treatment efficacy in multiple cancer types.^336^, ^337^ In addition, clinical studies supported the use of peposertib (formerly M3814) with desirable safety profile as monotherapy,^338^ but most ongoing clinical trials investigate its effects in combination with chemo‐ or radiotherapy in cancer. LY3023414 and CC‐115 are dual inhibitors that simultaneously target DNA‐PK and the mammalian target of rapamycin (mTOR), selectively blocking class I PI3K isoforms at low nanomolar concentrations.^339^, ^340^ CC‐115 was initially designed for mTOR, but was later reported to inhibit DNA repair and become particularly active in ATM‐deficient tumors.^341^ Recently Phase I trial on LY3023414 reported that LY3023414 was well tolerated as single agent in advanced cancers.^342^

## 5 DDR INHIBITOR‐BASED COMBINATION THERAPY

The combined treatment of DDR inhibitors with other treatment modalities including chemotherapy, radiotherapy, immunotherapy, or other targeted therapies. Moreover, recent data also supported the therapeutic value of concomitant targeting against nonredundant DDR components.^343^, ^344^ Here we summarized the ongoing combination trials on DDR inhibitors with chemotherapy, radiotherapy, target therapy (Table 2), with other DDR inhibitors (Table 3), and with immunotherapy (Table 4).

**TABLE 2:** Ongoing combination trials of DDR inhibitors with chemotherapy, radiotherapy, and target therapy

	Conditions	Interventions	Phase	Clinical trial*
Chemotherapy				
PARP				
	Cancer	Veliparib + VX‐970 + cisplatin	I	NCT02723864
	Metastatic breast cancer	Veliparib + carboplatin/paclitaxel	III	NCT02163694
	Ovarian, breast, pancreatic, prostate cancer	AZD5305 + Carboplatin/paclitaxe	I /II	NCT04644068
	Ovarian cancer	Veliparib + carboplatin/paclitaxel	III	NCT02470585
	Metastatic pancreatic adenocarcinoma	Veliparib + fluorouracil/irinotecan hydrochloride	II	NCT02890355
	SCLC	Veliparib + topotecan	I	NCT03227016
	Advanced solid tumors	IMP4297 + temozolomide	I	NCT04434482
	Triple negative breast cancer, ovarian cancer	KU‐0059436 (AZD2281) + carboplatin/paclitaxel	I	NCT00516724
	Breast cancer	ABT‐888 + temozolomide	II	NCT01009788
	Metastatic BRCA‐associated breast cancer	Veliparib + cisplatin	II	NCT02595905
	HR deficient advanced solid tumor malignancies	Niraparib + carboplatin	I	NCT03209401
	Prostate carcinoma	Niraparib + chemotherapy	II	NCT04592237
	Breast cancer	Olaparib + paclitaxel/carboplatin	II/III	NCT03150576
	Adrenal gland pheochromocytoma, paraganglioma	Olaparib + temozolomide	II	NCT04394858
	Advanced (stage IIIB‐C‐IV) ovarian, primary peritoneal and fallopian tube cancer	Rucaparib + paclitaxel/carboplatin	I /II	NCT03462212
	BRCA‐mutated ovarian carcinoma	Olaparib + chemotherapy	I	NCT03943173
	Gastric cancer	Olaparib + paclitaxel	II	NCT01063517
	Ovarian cancer	Olaparib + carboplatin/paclitaxel	II	NCT01081951
	Ovarian, fallopian tube, or primary peritoneal cancer	Rucaparib + chemotherapy	III	NCT02855944
	Recurrent solid tumors and ewing sarcoma	Talazoparib + onivyde	I /II	NCT04901702
	Uterine leiomyosarcoma	Olaparib + temozolomide	II	NCT03880019
	Ovarian cancer	Talazoparib + chemotherapy	III	NCT03642132
	Acute leukemia	Veliparib + temozolomide	I	NCT01139970
	Recurrent ovarian carcinoma	Niraparib + chemotherapy + atezolizumab	III	NCT03598270
	Metastatic malignant solid neoplasm	Veliparib + topotecan hydrochloride	I	NCT01012817
	IDH1 mutation	BGB‐290 + temozolomide	I/II	NCT03914742
	Recurrent glioma	Talazoparib + carboplatin	II	NCT04740190
	Refractory lymphomas undergoing stem cell transplant	Olaparib + chemotherapy	I	NCT03259503
ATM				
	Refractory cancer	AZD6738 + paclitaxel	I	NCT02630199
	Advanced cancer	ART0380 + gemcitabine	I/II	NCT04657068
ATR				
	Esophageal cancer	M6620 + cisplatin	I	NCT03641547
	Advanced stage solid tumors	BAY 1895344 + chemotherapy	I	NCT04514497
	Ovarian serous tumor	M6620 + gemcitabine	I	NCT02595892
	NSCLC, SCLC	VX‐970 (M6620) + topotecan	I/II	NCT02487095
	Cancer	AZD6738 + gemcitabine	I	NCT03669601
	Metastatic malignant solid neoplasm	M6620 + irinotecan hydrochloride	I	NCT02595931
	Refractory cancer	AZD6738 + paclitaxel	I	NCT02630199
	Advanced solid tumors	BAY 1895344 + cisplatin	I	NCT04491942
	Small cell cancers outside of the lungs	M6620 + topotecan	II	NCT03896503
CHK1				
	Brain tumor	LY2606368 + cyclophosphamide/gemcitabine	I	NCT04023669
WEE1				
	Metastatic pancreatic adenocarcinoma	MK‐1775 + paclitaxel/gemcitabine hydrochloride	I/II	NCT02194829
	Ovarian, primary peritoneal, or fallopian tube cancer	MK‐1775 + paclitaxel/gemcitabine hydrochloride	II	NCT02101775
Radiotherapy				
PARP				
	Triple negative breast cancer	Niraparib + radiation therapy/dostarlimab	II	NCT04837209
	Triple negative breast cancer	Niraparib + radiation therapy	I	NCT03945721
	Breast inflammatory carcinoma	Olaparib + radiation therapy	II	NCT03598257
	Malignant glioma without H3 K27M or BRAFV600 mutations	Veliparib + radiation therapy + temozolomide	II	NCT03581292
	Head and neck neoplasms	Olaparib + radiotherapy	I	NCT02229656
	Malignant gliomas	Temozolomide (TMZ) + radiotherapy	I/II	NCT03212742
ATM				
	Brain cancer	AZD1390 + radiation therapy	I	NCT03423628
	Advanced cancer	XRD‐0394 + palliative radiotherapy	I	NCT05002140
WEE1				
	Esophageal adenocarcinoma	Adavosertib + radiation therapy	I	NCT04460937
	Cervical carcinoma	Adavosertib + cisplatin/radiation therapy	I	NCT03345784
DNA‐PK				
	Rectal cancer	Peposertib + capecitabine/radiotherapy	I/II	NCT03770689
	Solid tumors	M3814 + radiotherapy	I	NCT03724890
	Advanced solid tumors	M3814 + fractionated RT/cisplatin	I	NCT02516813
	Glioblastoma, gliosarcoma	Nedisertib + radiation therapy/ temozolomide	I	NCT04555577
** **	Advanced solid tumor	XRD‐0394 + palliative radiotherapy	I	NCT05002140
Other target therapy				
PARP				
	BRCA1/2 gene mutated tumors	Niraparib + copanlisib (PI3Ki)	I	NCT03586661
	HER2 positive breast carcinoma	Niraparib + trastuzumab	I/II	NCT03368729
	Ovarian cancer	Olaparib + cediranib (VEGFR inhibitor)	N/A	NCT02681237
	Ovarian cancer patients	Niraparib + bevacizumab	II	NCT04734665
	Advanced solid tumors	Olaparib + CYH33 (PI3Kα inhibitor)	II	NCT04586335
	Breast cancer	Talazoparib + sacituzumab goviteca	I/II	NCT04039230
	Advanced breast carcinoma	Olaparib + cediranib(VEGFRi)	II	NCT04090567
	Metastatic breast cancer	Talazoparib + belinostat (HDACi)	I	NCT04703920
	Metastatic malignant solid neoplasm	Olaparib + onalespib (Hsp90 inhibitor)	I	NCT02898207
	Ovarian cancer	Niraparib + bevacizumab	I/II	NCT02354131
	High‐grade serous ovarian cancer	Olaparib + paclitaxel	II	NCT04261465
	Ovarian cancer	Olaparib + anlotinib (VEGFRi)	II	NCT04566952
	Breast cancer metastatic	Olaparib + vorinostat (HDACi)	I	NCT03742245
	Endometrial and ovarian cancer	Olaparib + AZD5363 (AKTi)	I/II	NCT02208375
	Metastatic prostate carcinoma, malignant neoplasm in the bone	Olaparib + cediranib (AZD‐2171) (VEGFRi)	II	NCT02893917
	EGFR‐mutated advanced lung cancer	Niraparib + osimertinib (EGFRi)	I	NCT03891615
	Ovarian cancer	Olaparib + cediranib	III	NCT03278717
	Advanced malignant solid neoplasm	Talazoparib tosylate + axitinib/ crizotinib (VEGFRi)	I	NCT04693468
	Endometrial serous adenocarcinoma	Olaparib + DS‐8201a (HER2i)	I	NCT04585958
	Ovarian cancer with no germline BRCA mutation	Olaparib + alpelisib (PIK3i)	III	NCT04729387
	Pancreatic cancer	Olaparib + cobimetinib (MEK/ERK inhibition)	I	NCT04005690
	Recurrent ovarian, primary peritoneal, or fallopian tube cancer	Olaparib + cediranib maleate	II	NCT02345265
	Recurrent ovarian, fallopian tube, or peritoneal cancer	Olaparib + cediranib maleate	I/II	NCT01116648
	Gastric or gastroesophageal junction cancer	Olaparib + ramucirumab (VEGFRi)	I/II	NCT03008278
	Metastatic NSCLC	Olaparib + cediranib	I	NCT02498613
	Ovarian, fallopian tube, or primary peritoneal cancer	Olaparib + cediranib maleate	II/II	NCT02502266
ATR				
	Chronic lymphocytic leukemia	AZD6738 + acalabrutinib (BTK inhibitor)	I/I	NCT03328273
Other treatments				
PARP				
	Neuroendocrine tumors	Talazoparib + 177Lu‐DOTA‐octreotate PRRT	I	NCT05053854
	Prostate cancer with ATM/BRCA1/2 gene mutation	Niraparib + radical prostatectomy	II	NCT04030559
	Prostate cancer	Olaparib + radium Ra223 dichloride	I	NCT03317392
	Neuroendocrine tumors, thymoma, mesothelioma	Olaparib + 177Lu‐DOTA‐TATE	I	NCT04375267
	Prostate carcinoma	Talazoparib + androgen deprivation therapy	II	NCT04734730
	Metastatic castration‐resistant prostate cancer	Rucaparib + Enzalutamide/zbiraterone	I	NCT04179396
	Prostate cancer	Talazoparib + enzalutamide	III	NCT04821622
ATR				
** **	SCLC, neuroendocrine cancers	Berzosertib + lurbinectedin	I/II	NCT04802174

* Data from https://clinicaltrials.gov.

**TABLE 3:** Ongoing combination trials of concomitant targeting against nonredundant DDR components

Combination	Conditions	Interventions	Phase	Clinical trial*
PARPi + ATRi				
	Advanced solid tumor	Talazoparib + RP‐3500	I	NCT04497116
	Advanced solid tumors (excluding prostate cancer)	Niraparib + BAY1895344	I	NCT04267939
	High‐grade serous carcinoma	Olaparib pill + AZD6738	II	NCT03462342
	Advanced solid tumor	Niraparib/Olaparib + RP‐3500	I/I	NCT04972110
	Gynaecological cancers	Olaparib + AZD6738	II	NCT04065269
	Cancer	AZD2281 + AZD5363 + AZD1775 + AZD6738	II	NCT02576444
	Advanced solid tumors	Niraparib + M1774	I	NCT04170153
	Malignant solid neoplasm	Olaparib + Ceralasertib	II	NCT03878095
	Recurrent ovarian, primary peritoneal, or fallopian tube cancer	Olaparib + Adavosertib	II	NCT03579316
	Prostate cancer	Olaparib + AZD6738	II	NCT03787680
	Clear cell renal cell carcinoma	AZD6738 + Olaparib	II	NCT03682289
	Advanced solid tumor	RP‐3500 + Niraparib/Olaparib	I/II	NCT04972110
PARPi + BETi				
	Advanced malignant solid neoplasm	Olaparib + Adavosertib	I	NCT04197713
	Ovarian cancer	Olaparib + Adavosertib	I	NCT04633239
	Triple negative breast cancer	Talazoparib + ZEN003694	II	NCT03901469
PARPi + CDK4/6i				
	Breast cancer	Niraparib + Abemaciclib	I	NCT04481113
PARPi + ATMi				
	Advanced solid tumours	Olaparib + AZD0156	I	NCT02588105
Other				
	Ovarian cancer	Olaparib + AsiDNATM	I/II	NCT04826198

* Data from https://clinicaltrials.gov.

**TABLE 4:** Ongoing combination trials of DDR inhibitors with immunotherapy

DDR	Conditions	Interventions	Phase	Clinical trial*
PARP				
	Endometrial neoplasms	Olaparib + durvaluma	II	NCT03951415
	Solid tumor	Rucaparib + atezolizumab	II	NCT04276376
	Biliary tract cancer	Rucaparib + nivolumab	II	NCT03639935
	Lung small cell carcinoma, neuroendocrine carcinoma	Niraparib + dostarlimab	II	NCT04701307
	Cervical cancer	Olaparib + pembrolizumab	II	NCT04483544
	Breast cancer	Olaparib + pembrolizumab	II	NCT03025035
	Ovarian, breast, gastric cancer, SCLC	Olaparib + durvalumab	I/II	NCT02734004
	Ovarian neoplasms	Niraparib + TSR‐042	II	NCT03574779
	Ovarian, fallopian tube, peritoneal cancer	Olaparib + tremelimumab	I/II	NCT02571725
	Metastatic pancreatic adenocarcinoma	Olaparib + pembrolizumab	II	NCT04548752
	Advanced malignant solid neoplasm	Niraparib + atezolizumab	I	NCT03830918
	Advanced malignant solid neoplasm	Olaparib + durvalumab/copanlisib	I	NCT03842228
	Metastatic breast carcinoma	Olaparib + atezolizumab	II	NCT02849496
	LSCL	Olaparib + durvalumab	I	NCT04728230
	Platinum‐sensitive ovarian cancer	OSE2101 + pembrolizumab	II	NCT04713514
	Advanced malignant solid neoplasm	Talazoparib + paclitaxel	I	NCT02317874
	Colorectal, breast neoplasms	Olaparib + durvalumab	I/II	NCT02484404
	Prostate carcinoma	Olaparib + durvalumab	II	NCT04336943
	Breast cancer	Niraparib + TSR‐042 (dostarlimab)	I	NCT04673448
	Triple negative breast cancer	Olaparib + durvalumab	II	NCT03167619
	Extensive SLSC	Talazoparib + atezolizumab	II	NCT04334941
	Fallopian tube mucinous adenocarcinoma	Olaparib + cediranib + durvalumab	II	NCT04739800
	Metastatic triple negative breast cancer	Olaparib + durvalumab	II	NCT03801369
	Breast, ovarian cancer	Niraparib + pembrolizumab	I/II	NCT02657889
	BRCAm ovarian, fallopian tube or primary peritoneal cancer	Olaparib + durvalumab/tremelimumab	II	NCT02953457
	Ovarian, fallopian tube, or primary peritoneal cancer	Rucaparib + nivolumab	III	NCT03522246
	Ovarian carcinosarcoma	Niraparib + TSR‐042 (dostarlimab)	II/III	NCT03651206
	Pancreatic adenocarcinoma	Niraparib + nivolumab/ipilimumab	I/II	NCT03404960
	Endometrial cancer	Olaparib + durvalumab	II	NCT03660826
	Metastatic solid tumors	Talazoparib + avelumab	II	NCT03330405
	BRCA1/2 and PALB2 mutated metastatic pancreatic cancer	Niraparib + dostarlimab	II	NCT04493060
	Advanced solid neoplasm	Veliparib + nivolumab	I	NCT03061188
	Metastatic melanoma with HR mutation	Olaparib + pembrolizumab	II	NCT04633902
ATM				
	Advanced solid tumors	Drug: BAY1895344 + pembrolizumab	I	NCT04095273
ATR				
	Advanced solid tumors	BAY1895344 + pembrolizumab	I	NCT04095273

* Data from https://clinicaltrials.gov.

### 5.1 Combinations with DNA‐damaging agents

#### 5.1.1 DDR inhibitor–chemotherapy combinations

As discussed, synergistic treatment of DDR inhibitors with cytotoxic chemotherapy has been performed, with schedules based on sequential chemotherapy administration followed by DDR inhibitor being proved clinically more beneficial and more tolerable.^145^, ^345^, ^346^, ^347^ The underlying mechanism for the synergy is that the rapidly dividing cancer cells are more likely to be affected by DNA damage directly caused by chemotherapy or indirectly from reactive oxygen species.^348^ For example, platinum derivatives (carboplatin, cisplatin, and oxaliplatin) produce intrastrand DNA cross‐links repaired by NER or the Fanconi anemia pathway.^349^ Antimetabolites result in stalling of the replication fork, whereas alkylating agents such as temozolomide lead to both single‐ and double‐ DNA strand breaks. Topoisomerase (Top) inhibitors include Top 1 inhibitors that generate SSBs, and Top 2 inhibitors that result in DSBs.^350^, ^351^ Meanwhile, epigenetics regulation also plays an important role in DDR, with the hypomethylation of DDR genes significantly associated with worse prognosis in glioblastoma patients.^352^ The epigenetics silencing of PRPF19 and TERT genes in glioblastoma cells overcomes their resistance to temozolomide treatment.^352^

Combining cytotoxic chemotherapies and PARP inhibitors has long been proposed based on the capability of PARP inhibitors to eliminate DNA lesions caused by chemotherapy. An early study suggested that a PARP inhibitor 3‐AB reversed tumor resistance to temozolomide (TMZ) in glioma models.^353^, ^354^ The combination of TMZ and PARP inhibitor NU1025 was later found to suppress tumor growth and improve overall survival of central nervous system lymphoma.^355^ These successful preclinical results urged the clinical evaluation of the TMZ/PARPi combination in patients with advanced gliomas, where the combination regimen demonstrated modest antitumor efficacy and overall tolerability.^356^ A randomized Phase II/III study (NCT02152982) investigated the combination of PARPi veliparib and TMZ, which improved disease outcome in tumors with MGMT promoter hypermethylation.^357^ Interestingly, the combination was previously found to be ineffective in MGMT‐unmethylated cell lines, suggesting the predicting value of MGMT promoter methylation status in tumor response to TMZ/veliparib combination therapy.^357^ The combination was further tested in other cancer types (NCT01009788, NCT01638546), but failed to induce significant survival benefits in patients with small cell lung cancer.^358^ Early PARP inhibitors 3‐AB and PJ34 were shown to overcome tumor resistance to cisplatin in several cancer types,^359^, ^360^, ^361^ and olaparib was later suggested to enhance the therapeutic effect of cisplatin in lung cancer cells.^362^, ^363^ These preclinical success allowed the initiation of clinical studies on olaparib in patients with platinum‐sensitive ovarian cancer (NCT01081951), where olaparib increased PFS in patients receiving platinum/paclitaxel monotherapy, but failed to improve overall survival.^65^, ^347^ The combination of PARP inhibitor veliparib with carboplatin and paclitaxel was tested in patients with triple‐negative breast cancer patients (NCT02032277) but did not bring survival benefits.^364^

The ATR inhibitor M6620 demonstrated strong efficacy in combination with cisplatin, which later entered clinical trial and resulted in objective responses in clinical trial either as single agent or cotherapy with carboplatin.^365^, ^366^ Other DDR inhibitors used along with definitive chemotherapy are underway, including DNA‐PK inhibitor M9831, the Phase I evaluation of which was completed in 2019 to determine the maximum tolerated dose of M9831 and its efficacy with or without doxorubucin in advanced cancer patients (NCT02644278).

#### 5.1.2 DDR inhibitor–radiotherapy combinations

The systematic delivery of chemotherapy poses a challenge to its the combinatorial therapy with DDR inhibitors. The overlapping toxicities, predominantly myelosuppression, have led to the termination of many clinical trials.^367^, ^368^ To date, DNA‐damaging agents still remain the mainstay of nonoperative cancer treatment, and besides chemotherapy, radiation therapy is an optional treatment. The ionization effect of radiation producing oxygen free radicals causes DNA damage in cells with 1 Gy of ionizing radiation being able to generate 1000 SSBs and 35 DSBs.^369^ While radiation has been proved effective by accumulating evidence in combating tumors, an important question is how to reduce the amount of radiation delivered to normal tissues and thus prevent the acute and chronic toxicities. A strategy to intensify the efficacy and at the same time reduce toxicity of radiotherapy is the combination with novel targeted therapies, which increases the radiosensitivity of cancer cells to a greater extent than normal cells.^370^ Given that radiation causes different DNA lesions including base damaging, SSBs, and DSBs, the simultaneous inhibition of key DDR enzymes thus becomes a promising strategy.^371^ Furthermore, the clear correlation between radioresistance and increased DNA repair capacities further justify the combinational use of radiotherapy and DDR inhibition.^372^ However, early efforts on DDR blockade such as PARP inhibitors failed to achieve consistent results.^373^, ^374^, ^375^ The suboptimal synergistic effect might be attributed to the fact that DSBs caused by conventional radiation are repaired predominately through the NHEJ pathway, rather than PARP‐regulated BER pathway. Moreover, compared with conventional photon‐based radiation, HR repair pathway is more engaged in the repair of heavy ion (carbon and iron)‐induced DNA damage.^375^, ^376^ The radiosensitization approaches include inhibitors that prevent S and G2/M cell‐cycle arrest that allows DNA damage repair, such such as PARP, CHK1, WEE1, ATR, and DNA‐PK inhibitors.

VE‐821 is a ATR inhibitor with potent inhibitory activities on the phosphorylation of H2AX and CHK1 by ATR, and sensitizing effect on cancer cells to radiotherapy and genotoxic chemotherapeutics.^66^, ^231^, ^377^, ^378^, ^379^ Notably, the radiosensitization of VE‐821 was even more profound in hypoxic cells.^377^ M6620 (VX‐970) is the improved analogue of VE‐821 and its synergistic potential with radiotherapy has been widely studied in preclinical settings.^232^ In esophageal cancer, M6620 was shown to enhance radiation‐induced tumor growth arrest both in vitro and in vivo.^380^, ^381^ The concurrent treatment of M6620 and radiation was recently reported to improve the overall survival in mouse models, supporting the ongoing clinical trial (NCT02589522) assessing the sensitizing effects of M6620 to whole brain irradiation in NSCLC patients with brain metastases.^382^ AZD6738 was intensively investigated in various cancers, especially ATM‐deficient cancers as a monotherapy; recent attempt has converged on its combination therapies.^286^, ^383^, ^384^, ^385^ The multiparametric cell‐based assays measuring DNA damage and cell‐cycle transition are induced by the treatment of AZD6738, and the in vivo mouse xenograft studies provide strong rationale for the design of Phase I clinical trials.^386^ The accumulating promising results from preclinical studies encouraged the assessment of AZD6738 in more than 25 clinical trials including monotherapies in hematological malignancies (NCT01955668, NCT03770429) and in refractory solid tumors (NCT02223923, NCT03022409), and in combination with radiotherapy (NCT02223923).

WEE1 is involved in the initiation of G2 checkpoint, and the inhibition of Wee1 would subsequently cause unscheduled mitotic entry and increased replication stress.^281^ It has been reported that increased sensitivity to WEE1 inhibition through mechanisms outside of cell‐cycle checkpoint defects, such as DDR aberrations and nucleotide resource starvation, with single‐agent activity observed even in TP53‐wild‐type cancer cells.^387^, ^388^, ^389^, ^390^ The critical role of p53 in the regulation of G1 checkpoints provides a strong rationale for the use of WEE1 inhibitors in p53‐deficient cells.^391^ A WEE1 inhibitor, adavosertib (AZD1775 or MK‐1775), was shown to sensitize p53‐deficient cells to DNA‐damaging radiotherapy via the induction of mitotic lethality.^281^, ^392^ Thus, recent clinical development has focused to the concurrent treatment of WEE1 inhibitors and DNA‐damaging treatments such as radiation therapy in TP53 mutant tumors. Following the evaluation of Phase I study as single agent,^393^ AZD1775 has demonstrated overall survival benefits when combining radiation in patients with advanced pancreatic cancer.^290^

As NHEJ is the predominant pathway for the repair of traditional radiotherapy,^394^ the specific targeting of NHEJ by DNA‐PK inhibitors is thus considered as a potential combination partner for radiation. Currently, three DNA‐PK inhibitors are under clinical trials: M9831 (VX‐984), nedisertib (M3814), and CC‐115. In addition to monotherapy, CC‐115 is now being investigating in combination with androgen‐deprivation therapy (ADT) in castrate‐resistant prostate cancer patients (NCT02833883) and with radiation in glioblastoma patients (NCT02977780). Inspired by results from a Phase I trial involving patients with tumors in the head and neck or thorax,^395^ a growing number of trials are underway to assess the efficacy of nedisertib monotherapy or with radiation.

### 5.2 DDR inhibitor–DDR inhibitor combinations

The initial purpose of cotargeting key DDR elements was to overcome the acquired resistance to a single DDR inhibitor, predominantly PARP inhibitors. In the light of the variety of DNA repair mechanisms, the combination of one or more of DDR inhibitors to induce synthetic lethality is biologically applicable, even in HR‐proficient cells.^396^ An exciting example was the coinhibition of PARP and WEE1 inhibitor. The combination of adavosertib and olaparib synergistically promoted radiosensitivity of pancreatic cancer cells by impairing their HR repair capacity to achieve synthetic lethality, which led to the initiation of multiple clinical trials (NCT02723864, NCT02576444, and NCT02511795).^397^ In PARPi‐resistant cells with SLFN11 deficiency, the additional ATR inhibition would overcome the resistance due to the fact that SLFN11‐inactive cells were more reliant on the ATR pathway for DNA repair.^398^ Likewise, ATR blockade further disrupted HR repair pathway in BRCA‐deficient cancer cells.^399^ In lymphoma models, ATR inhibitor AZD6738 displayed a strong synergistic cytotoxic effect when combined with Chk1 inhibtor or WEE1 inhibitor, further expanding the repertoire of DDR–DDR therapeutic combinations.^400^

In addition to ATR, HR‐deficient tumor cells are also increasingly reliant on other alternative repair pathways such as a type of a‐EJ, named microhomology‐mediated end joining (MMEJ) for survival, suggesting the potential of cotargeting PARP and key members of MMEJ.^21^ Other combination partners for PARP inhibitors include the antagonists of PI3K‐AKT pathway^401^ and BRD4 protein, which has been shown to downregulate several DDR genes and increase the sensitivity of HR‐proficient tumors to PARP inhibition.^402^, ^403^, ^404^ Previous work shows that recently, the combined inhibition of PARP1 and DNA‐PK was found to suppress HNSCC tumor growth in vitro and in vivo compared to either agent used alone.^405^ The underlying mechanism may be the cooperation between PARP1 and DNA‐PK_cs_ to recruit XRCC1 to mediate DNA repair.^406^, ^407^, ^408^

### 5.3 DDR inhibitor–immunotherapy combinations

**FIGURE 3:**
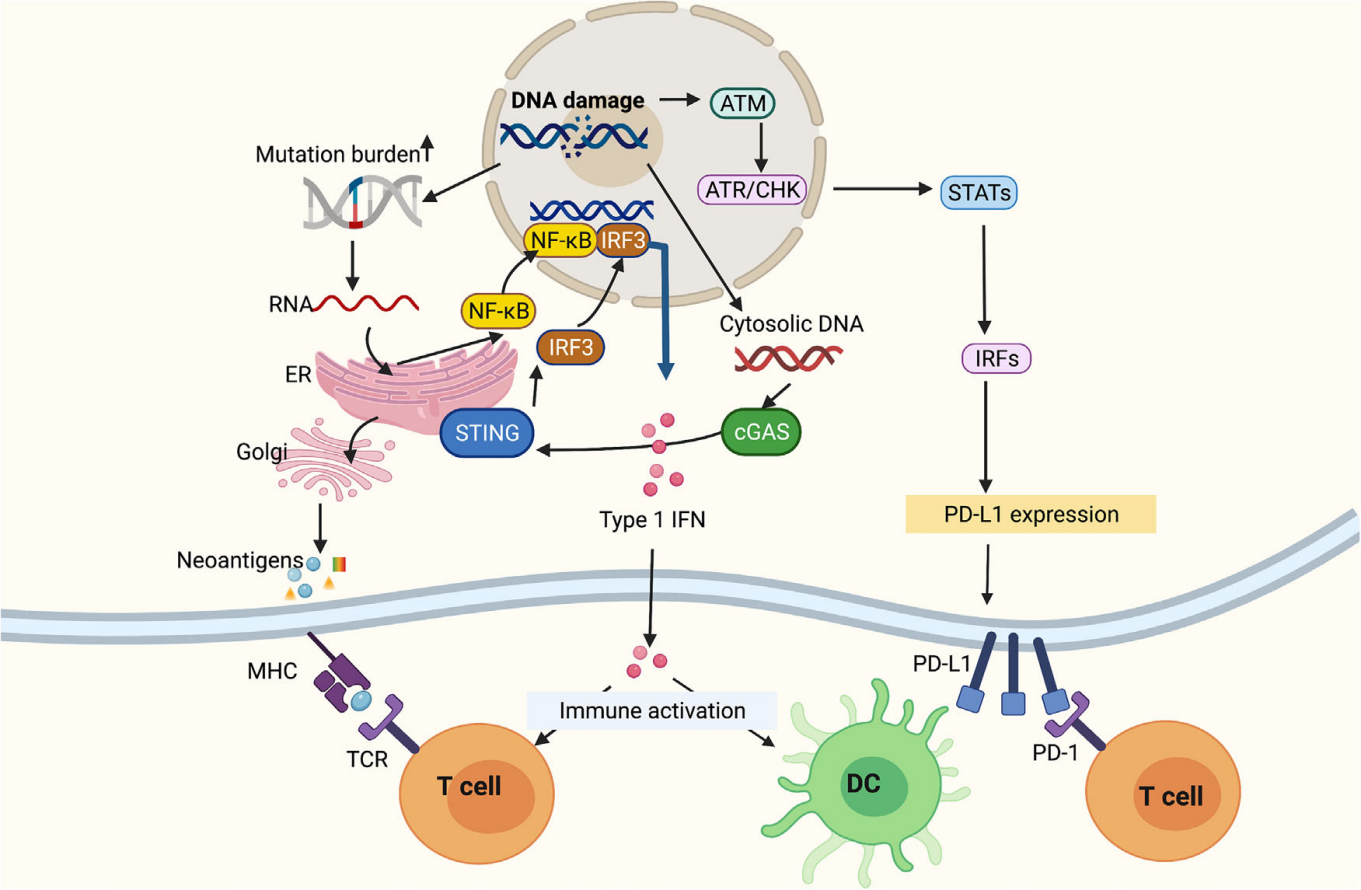
The interaction between DNA damage with immune responses. The activated STING pathway leads to upregulation of type I IFNs, which enhances the cross‐presentation of dendritic cells (DCs) and T‐cell activation. Unrepaired DNA damage may generate tumor neoantigens and thereby improving tumor recognition by T cells. However, DNA damage or DDR deficiencies have also been shown to upregulate PD‐L1 expression. cGAS, cyclic GMP–AMP synthase; DDR, DNA damage response; DC, dendritic cell; DSB, double‐strand break; ER, endoplasmic reticulum; IRF3, interferon regulatory factor 3, IFN, interferon; NF‐κB, nuclear factor kappa‐B; STING, stimulator of interferon genes; TBK1, TANK‐binding kinase 1. Figure was created with bioRender

The alteration in immune environment caused by DDR deficiency may be used to facilitate the sensitization of immunotherapies.^409^ Deficient DDR results in accumulated DNA damage in cells and increases their mutational burden, particularly in tumor cells that normally experience high level of endogenous or exogenous DNA damage. It is becoming increasingly clear that DNA damage could induce the production of immune‐regulatory cytokines such as type I IFNs.^410^, ^411^, ^412^ DNA normally resides in the nucleus or mitochondria, and once it is released to the cytoplasm, it triggers a series of immune response. DNA binds to cyclic guanosine monophosphate (GMP)–adenosine monophosphate (AMP) synthase (cGAS), which leads to the conformational change of the catalytic subunit of cGAS allowing the formation of the second messenger cyclic GMP–AMP (cGAMP).^413^ cGAMP then activates STING and its downstream transcription factors IRF3 and NF‐κB via kinases TBK1 and IKK, respectively. As shown in Figure 3, IRF3 and NF‐κB then translocate into the nucleus and induce the expression of multiple cytokines such as IFNs. DDR dysfunction or the combination therapy with DDR inhibitors further enhances DNA damage, which when transfers into cytosolic DNA and triggers the stimulator of interferon genes (STING) pathway to activate innate immune responses.^414^, ^415^

Tumors harboring mutations in BRCA1/2 or ATM were identified with high level of cytosolic DNA, which stimulated the innate immune activities and correlated with a durable response to ICIs.^416^, ^417^ In addition, the induced neoantigens of tumor cells could stimulate the host immune response including the intratumoral infiltration of CD8+ T cells, which have long been characterized as a predictive marker for cell response to ICIs.^418^, ^419^, ^420^ Recent evidence suggested that deleterious DDR‐related gene mutations are a frequent event in NSCLC, which indicates improved clinical outcomes in NSCLC patients with PD‐(L)1 antibody treatment.^421^ Thus, it is conceivable that DDR inhibitors may be able to convert immunologically “cold” into “hot” tumors and sensitize tumor cells to ICIs.^422^, ^423^ A growing number of clinical trials evaluating this drug combination in cancer patients are underway.^93^ Figure 3 presents a simplified scheme of the interaction between DNA damage with immune responses.

PARP inhibitors are one of the most extensively studied DDR inhibitors in clinical development and in the context of synthetic lethality such as cells with BRCA1/2 mutations, PARP inhibition is considered proinflammatory.^424^ Cells treated with PARP inhibitors exhibited an increased level of PD‐L1 expression, supporting the concomitant use of PARP inhibitors and ICIs.^423^ Interestingly, cancer stem cells (CSCs) displayed higher expression of PD‐L1 compared to nonstem cell cancer cells, which might contribute to the long‐term survival improvement by immunotherapy^425^ and make ICIs a potential strategy to overcome resistance of CSCs to PARP inhibitors.^426^ However, PARP inhibition has recently been shown to attenuate immune response in mice by suppressing thymocyte maturation.^427^ It is thus intriguing to speculate whether toxicity of ICIs could be reduced when used in combination with PARP inhibitors.

CDK4/6 inhibitors could convert HR into NHEJ mechanism in cells treated with ionizing radiation in several tumor models,^428^, ^429^, ^430^ which was likely attributed to the active involvement of cyclin D‐CDK4/6‐RB pathway in DDR.^431^ Besides their radiosensitization effects, CDK4/6 inhibitors were also reported to reduce the T‐cell exclusion and immune evasion in ICI‐resistant melanoma cells.^432^ It is therefore not surprising that the combination of CDK4/6 inhibitors and anti‐PD‐L1 therapy led to substantial tumor regression in xenograft mouse models.^433^, ^434^ Clinical trials sought to determine the efficacy of FDA‐approved CDK4/6 inhibitors such as palbociclib and abemaciclib combined with pembrolizumab in patients with HR‐positive breast cancer (NCT02779751, NCT02778685), where the drug combination induced a higher objective response rate than either monotherapy and later entered clinical trials on other cancer types.^435^

Other combination partner for ICI includes the CHK1 inhibitor prexasertib (LY2606368), which potently activated the STING/TBK1/IRF3 innate immune pathway and upregulated tumor expression of PD‐L1, suggesting its synergistical potential with ICIs.^436^, ^437^ Several action mechanisms of the combination therapy have been proposed. For example, ATR inhibitor (BAY1895433) targeting the ATR‐CHK1 signaling could activate CDK1‐SPOP axis, wchich results in the destabilization of PD‐L1, proving a strong rationale for the concomitant use of ATRi with anti‐PD‐L1 therapy.^438^ Adavosertib is currently the only WEE1 inhibitor under clinical trials and its combination with anti‐PD‐L1 monoclonal antibody durvalumab is under assessment in a Phase I trial (NCT02617277).^439^

## 6 REMAINING CHALLENGES AND FUTURE PERSPECTIVES

Cell response to DNA damage is a complex process involving various signal networks and proteins, which are differentially activated or inactivated in specific cancer types. For instance, breast, ovarian, and bladder cancers are likely accompanied with alterations in HR genes, whereas some gastric and colorectal tumor subgroups present a hypermutator phenotype lacking aneuploidy. Furthermore, the DNA repair capacity also varies among different cell types. For example, the repair efficiency of human embryonic stem cells is the higher than differentiated cell types,^440^ and some tumor cells present upregulated damage repair such as the high level of MGMT repair activity in gliomas.^441^, ^442^ Thus, characterization of every single type of tumor to identify its specific profile of deregulated DDR components will facilitate personalized treatment of cancer patients. Next‐generation sequencing provides an opportunity for precision medicine by analyzing the whole‐genome alterations associated with DNA repair across different cancer types. It is recently found through next‐generation sequencing that epigenetic regulators also appear to play a particularly important role in cancer events.^443^ For example, epigenetic silencing of genes leads to loss‐of‐function events of DDR proteins.

The initial idea for the DDR inhibitor‐based combination therapy was to enhance the efficacy of conventional treatments. Although DDR inhibitors have been widely conducted on unselected patients, recent research interest tends to use these drug combinations in tumors with specific genetic backgrounds such as p53 mutation and BRCA alterations, which make cells more susceptible to DDR inhibitors. Emerging clinical trials are ongoing to explore the potential predictive markers for patients’ response to combinational therapy, including alterations in genes such as ATM, BRCA1, BRCA2, CDK12, CHEK1, MYC, PARP1, PIK3CA, and PTEN (NCT03842228, NCT02546661).

The early knowledge that DNA repair deficiency leading to increased neoantigen and tumor mutational load makes ICI a potential combination partner for DDR inhibitors. However, high mutational burden is a not a guarantee for efficient ICI response, given the varying level of immunogenicity induced by different DNA repair–deficient backgrounds. The immune score and mutational signature have been proved feasible in evaluating the response of ovarian cancer patients to niraparib and pembrolizumab.^444^ Reliable predictive biomarkers are needed to identify the specific subset of patients responsive to ICI and DDR inhibitor combinations.^445^ One such strategy is to integrate indexes from multiple platforms, such as combining tumor mutational burden with immune activity marker. The immune activity can be reflected by intratumor immune infiltrations and STING pathway.^446^

Targeting methylation pathways is a promising anticancer strategy.^447^ Accumulating evidence has suggested the epigenetics regulation on DDR. Multiple histone methyltransferases and demethylases have been described to facilitate chromatin remodeling and chromatin‐based DDR activities. However, mechanisms of how histone methylation is involved in DDR remains to be elucidated. Given the correlation between PARP and histone methylation, identifying the involvement of methylation signaling in DDR would bring new therapeutic approaches for cancer treatment.

Finally, the increased replication stress and DNA repair defects in tumors provide a therapeutic opportunity that makes cancer cells more vulnerable to DDR inhibition than normal cells. However, the rapid development of clinical DDR inhibitors has raised a concern on toxicity, which is frequently accompanied with other anticancer therapies. It is rather imperative to identify optimal doses, combinations, and schedules of DDR inhibitors to minimize their adverse effects and more ideally, enhance the efficacy. It has to be addressed that DDR proteins initially possess essential physiological functions that recognize and fix DNA damage in normal cells, the repression of which may be deleterious due to the increased mutagenic load in normal tissues. Surveillance on long‐term toxicity of DDR inhibition may thus be added into clinical trial design.

## CONFLICT OF INTEREST

The authors declare no conflict of interests.

## AUTHOR CONTRIBUTIONS

Wang Manni offered the main direction and significant guidance of this manuscript. Wang Manni, Siyuan Chen and Danyi Ao drafted the manuscript and illustrated the figures for the manuscript.

## ETHICS APPROVAL

Not Applicable

## Data Availability

The authors confirm that the data supporting the findings of this study are available within the review.
